# Genomic and phenotypic insights into the expanding phylogenetic landscape of the *Cryptococcus* genus

**DOI:** 10.1101/2025.06.18.660340

**Published:** 2025-06-24

**Authors:** Marco A. Coelho, Márcia David-Palma, Andrey M. Yurkov, Joseph Heitman

## Abstract

The fungal genus *Cryptococcus* includes several life-threatening human pathogens as well as diverse saprobic species whose genome architecture, ecology, and evolutionary history remain less well characterized. Understanding how some lineages evolved into major pathogens remains a central challenge and may be advanced by comparisons with their nonpathogenic counterparts. Integrative approaches have become essential for delimiting species and reconstructing evolutionary relationships, particularly in lineages with cryptic diversity or extensive chromosomal rearrangements. Here, we formally characterize six *Cryptococcus* species representing distinct evolutionary lineages, comprising both newly discovered and previously recognized but unnamed taxa, through a combination of phylogenomic analyses, divergence metrics, chromosomal comparisons, mating assays, and phenotypic profiling. Among pathogenic taxa, we formally name *Cryptococcus hyracis* sp. nov., corresponding to the previously characterized VGV lineage within the *C. gattii* complex. In parallel, we describe five saprobic, nonpathogenic species isolated from fruit, soil, and bark beetle galleries, spanning four phylogenetic clades. We identify a strong ecological association with bark beetles for *Cryptococcus porticicola* sp. nov., the only newly described nonpathogenic species with multiple sequenced strains from diverse sites. In this species, we detect strain-level chromosomal variation and evidence of sexual reproduction, along with population-level signatures of recombination consistent with ongoing genetic exchange. Across the genus, chromosome-level comparisons reveal extensive structural variation, including species- and strain-specific rearrangements that may restrict gene flow. We also identify multiple instances of chromosome number reduction, often associated with centromere inactivation following interchromosomal rearrangements. Comparative metabolic profiling with Biolog phenotype microarrays reveals clade-level differentiation and distinct substrate preferences, which may reflect metabolic divergence and habitat-specific diversification. Notably, we confirm that thermotolerance is restricted to clinically relevant taxa. These findings refine the species-level taxonomy of *Cryptococcus* , broaden its known genomic and ecological diversity, and strengthen the framework for investigating speciation, adaptation, and the emergence of pathogenicity within the genus.

## Full Text Availability

The license terms selected by the author(s) for this preprint version do not permit archiving in PMC. The full text is available from the preprint server.

