# Genomic and phenotypic insights into the expanding phylogenetic landscape of the *Cryptococcus* genus

**DOI:** 10.1101/2025.06.18.660340

**Published:** 2025-07-30

**Authors:** Marco A. Coelho, Márcia David-Palma, Aleksey V. Kachalkin, Miroslav Kolařík, Benedetta Turchetti, José Paulo Sampaio, Michael J. Wingfield, Matthew C. Fisher, Andrey M. Yurkov, Joseph Heitman

**Affiliations:** 1 Department of Molecular Genetics and Microbiology, Duke University Medical Center, Durham, North Carolina, USA; 2 M.V. Lomonosov Moscow State University, Moscow, Russia; 3 G.K. Skryabin Institute of Biochemistry and Physiology of Microorganisms, PSCBR RAS, Pushchino, Russia; 4 Laboratory of Fungal Genetics and Metabolism, Institute of Microbiology of the Czech Academy of Sciences, Vídeňská 1083, Prague, Czechia; 5 Department of Agricultural, Food and Environmental Sciences, University of Perugia, Perugia, Italy; 6 UCIBIO, i4HB, Departamento de Ciências da Vida, Faculdade de Ciências e Tecnologia, Universidade Nova de Lisboa, Caparica, Portugal; 7 Department of Biochemistry, Genetics and Microbiology, Forestry and Agricultural Biotechnology Institute (FABI), University of Pretoria, Pretoria, South Africa; 8 Department of Infectious Disease Epidemiology, Imperial College London, London, United Kingdom; 9 Leibniz Institute DSMZ-German Collection of Microorganisms and Cell Cultures, Braunschweig, Germany

**Keywords:** Fungal speciation, human fungal pathogens, comparative genomics, chromosome evolution, rRNA architecture, digital DNA–DNA hybridization

## Abstract

The fungal genus *Cryptococcus* includes several life-threatening human pathogens as well as diverse saprobic species whose genome architecture, ecology, and evolutionary history remain less well characterized. Understanding how some lineages evolved into major pathogens remains a central challenge and may be advanced by comparisons with their nonpathogenic counterparts. Integrative approaches have become essential for delimiting species and reconstructing evolutionary relationships, particularly in lineages with cryptic diversity or extensive chromosomal rearrangements. Here, we formally characterize six *Cryptococcus* species representing distinct evolutionary lineages, comprising both newly discovered and previously recognized but unnamed taxa, through a combination of phylogenomic analyses, divergence metrics, chromosomal comparisons, mating assays, and phenotypic profiling. Among pathogenic taxa, we formally name *Cryptococcus hyracis* sp. nov., corresponding to the previously characterized VGV lineage within the *C. gattii* complex. In parallel, we describe five saprobic, nonpathogenic species isolated from fruit, soil, and bark beetle galleries, spanning four phylogenetic clades. We identify a strong ecological association with bark beetles for *Cryptococcus porticicola* sp. nov., the only newly described nonpathogenic species with multiple sequenced strains from diverse sites. In this species, we detect strain-level chromosomal variation and evidence of sexual reproduction, along with population-level signatures of recombination consistent with ongoing genetic exchange. Across the genus, chromosome-level comparisons reveal extensive structural variation, including species- and strain-specific rearrangements that may restrict gene flow. We also identify multiple instances of chromosome number reduction, often associated with centromere inactivation following interchromosomal rearrangements. Comparative metabolic profiling with Biolog phenotype microarrays reveals clade-level differentiation and distinct substrate preferences, which may reflect metabolic divergence and habitat-specific diversification. Notably, we confirm that thermotolerance is restricted to clinically relevant taxa. These findings refine the species-level taxonomy of *Cryptococcus*, broaden its known genomic and ecological diversity, and strengthen the framework for investigating speciation, adaptation, and the emergence of pathogenicity within the genus.

## Author Summary

*Cryptococcus* is a genus of fungi that includes species capable of causing life-threatening infections in humans, as well as many other species that inhabit soil, fruit, decaying wood, and insect-associated environments with no known links to disease. Here, we formally describe six previously unnamed species based on genome-wide comparisons, genetic divergence metrics, chromosome structure, reproductive behavior, and metabolic profiling. One of these species belongs to the pathogenic lineage, while the others are distinct environmental species, including several recovered from bark beetles or their galleries, indicating repeated occurrence with this ecological niche. For one of these insect-associated species, represented by multiple isolates, we detected signatures of sexual reproduction and ongoing genetic exchange in natural populations. This study expands the framework for investigating how certain *Cryptococcus* lineages adapt to new environments or acquire traits relevant to pathogenicity. It also underscores the importance of continuous environmental sampling, which remains essential for capturing fungal diversity, and illustrates the power of combining genomic and phenotypic data to refine species boundaries and uncover lineages that may otherwise remain undetected.

## Introduction

The basidiomycete genus *Cryptococcus* was formally established by Vuillemin in 1901 to describe encapsulated, non-fermentative yeasts that lacked ascospore formation—a key distinction from the genus *Saccharomyces* [1, 2]. This taxonomic revision unified two independent discoveries made in 1894: German physicians Otto Busse and Abraham Buschke had isolated a *Saccharomyces*-like yeast from a tibial lesion in a young woman with a chronic granulomatous infection [3], while Italian microbiologist Francesco Sanfelice recovered a similar organism from peach juice, naming it *Saccharomyces neoformans* based on its distinctive colony morphology [4]. Vuillemin’s renaming of the organism as *Cryptococcus neoformans* formalized the genus and established its association with human disease.

Over the following decades, *C. neoformans* became recognized as the causative agent of cryptococcal meningoencephalitis, a life-threatening fungal infection with tropism for the central nervous system, particularly in immunocompromised individuals [5]. Its global burden escalated during the HIV/AIDS pandemic of the 1980s and 1990s, when it became a common AIDS-defining illness [2]. Early classifications relied on phenotypic traits such as capsule morphology and serological markers, which were valuable for identification but lacked resolution to resolve cryptic diversity or deeper evolutionary relationships.

A major advance came in 1975, when K.J. Kwon-Chung successfully induced the sexual state of *C. neoformans* (serotype D, now *C. deneoformans*), leading to the description of the teleomorph *Filobasidiella neoformans* [6]. The observed basidia resembled those of the genus *Filobasidium*, prompting the proposal of a new genus to accommodate the sexual state. However, subsequent revisions to the International Code of Nomenclature of algae, fungi and plants (ICNafp)—which moved toward unifying asexual and sexual names—led to the synonymization of *Filobasidiella* under the older name *Cryptococcus*, which has nomenclatural priority. The discovery of the sexual cycle not only clarified the reproductive biology of this organism but also emphasized the ecological and clinical relevance of spore production, which facilitates environmental dispersal and likely exposure of mammalian hosts [7].

Today, *Cryptococcus* is best known for its emblematic pathogenic species, particularly *C. neoformans* and *C. gattii*, which together cause over 112,000 deaths annually, primarily among individuals with advanced HIV/AIDS, with the highest burden in sub-Saharan Africa [5, 8]. Owing to their substantial global impact, *C. neoformans* and *C. gattii* have been designated as critical- and medium-priority pathogens, respectively, on the WHO fungal priority list [9]. These two species belong to a broader pathogenic clade presently comprising seven recognized human pathogens: *C. neoformans*, *C. deneoformans*, and five members of the *C. gattii* complex (*C. gattii*, *C. bacillisporus*, *C. deuterogattii*, *C. tetragattii*, and *C. decagattii*) [10]. These taxa differ in virulence, antifungal susceptibility, geographic distribution, and host susceptibility, differences increasingly illuminated through comparative genomics and population-level studies. For instance, *C. deuterogattii* and *C. gattii* sensu stricto frequently cause disease in immunocompetent individuals, the former species being responsible for a well-documented outbreak on Vancouver Island that later expanded to mainland Canada and the Pacific Northwest of the United States [11, 12]. In contrast, *C. neoformans*, *C. deneoformans*, *C. bacillisporus*, *C. tetragattii*, and *C. decagattii* are more often associated with infections in immunocompromised hosts [10, 13, 14]. A recently described, deeply divergent lineage within the *C. gattii* complex (VGV) has not yet been linked to human disease but exhibits thermotolerance and other traits suggestive of pathogenic potential [15].

As taxonomic resolution improves, formally naming new *Cryptococcus* species has become both necessary and important. While concerns have been raised about the added complexity for clinical practice [16], failure to recognize distinct phylogenetic lineages can impede diagnosis and obscure clinical and ecological patterns. Even within the *C. gattii* complex, species differ in virulence, antifungal susceptibility, geographic range, and host susceptibility (i.e., immunocompetent vs. immunocompromised) [10, 13, 14], reinforcing the need for accurate species-level distinctions in both public health and evolutionary research.

Beyond the pathogenic species, *Cryptococcus* harbors several nonpathogenic taxa. Some have been recognized for decades—including *C. wingfieldii*, *C. amylolentus*, *C. depauperatus*, and *C. luteus* [17–20]—while others, such as *C. floricola* [21], have been more recently described. These species are known exclusively from environmental isolates, typically recovered from soil, plants, or insects, and are presumed saprobic, although *C. depauperatus* and *C. luteus* have been proposed as potential mycoparasites [22–24]. These taxa fall into two distinct clades: one comprising *C. amylolentus*, *C. floricola*, and *C. wingfieldii*, and another represented by *C. depauperatus*. The phylogenetic placement of *C. luteus* remains unresolved due to the lack of genome sequence data. Phylogenetically, the closest known relatives to *Cryptococcus* are found in the genus *Kwoniella*, with both genera forming a well-supported clade within the *Tremellales* (class *Tremellomycetes*) [19, 25].

Building on this foundation, recent work and database mining have revealed additional deeply divergent *Cryptococcus* lineages that remain undescribed [25–27]. High-quality genome assemblies from these lineages have enabled broader comparative analyses of gene content, chromosomal organization, and mating-type locus (*MAT*) structure, offering new insights into the genomic basis of ecological adaptation and potential pathogenicity [25, 27]. Here, we build upon and expand these genomic efforts by integrating newly sequenced genomes with existing data to formally describe six new *Cryptococcus* species representing deeply diverging lineages across multiple clades. Through genome-scale phylogenies, divergence metrics, and chromosomal structural comparisons, we analyze species boundaries, identify evidence of sexual reproduction within a previously uncharacterized bark beetle-associated species, and uncover lineage-specific reductions in chromosome number. We further document phenotypic differentiation across clades, including variation in substrate assimilation profiles, thermotolerance, and melanin production. In doing so, we combine the biological and phylogenetic species concepts to establish a stable, well-supported classification within the genus *Cryptococcus* and provide a broader framework for exploring ecological adaptation and the evolutionary emergence of pathogenicity.

## Results

### Species phylogeny reveals novel *Cryptococcus* diversity and deep evolutionary divergence

**Fig 1.**
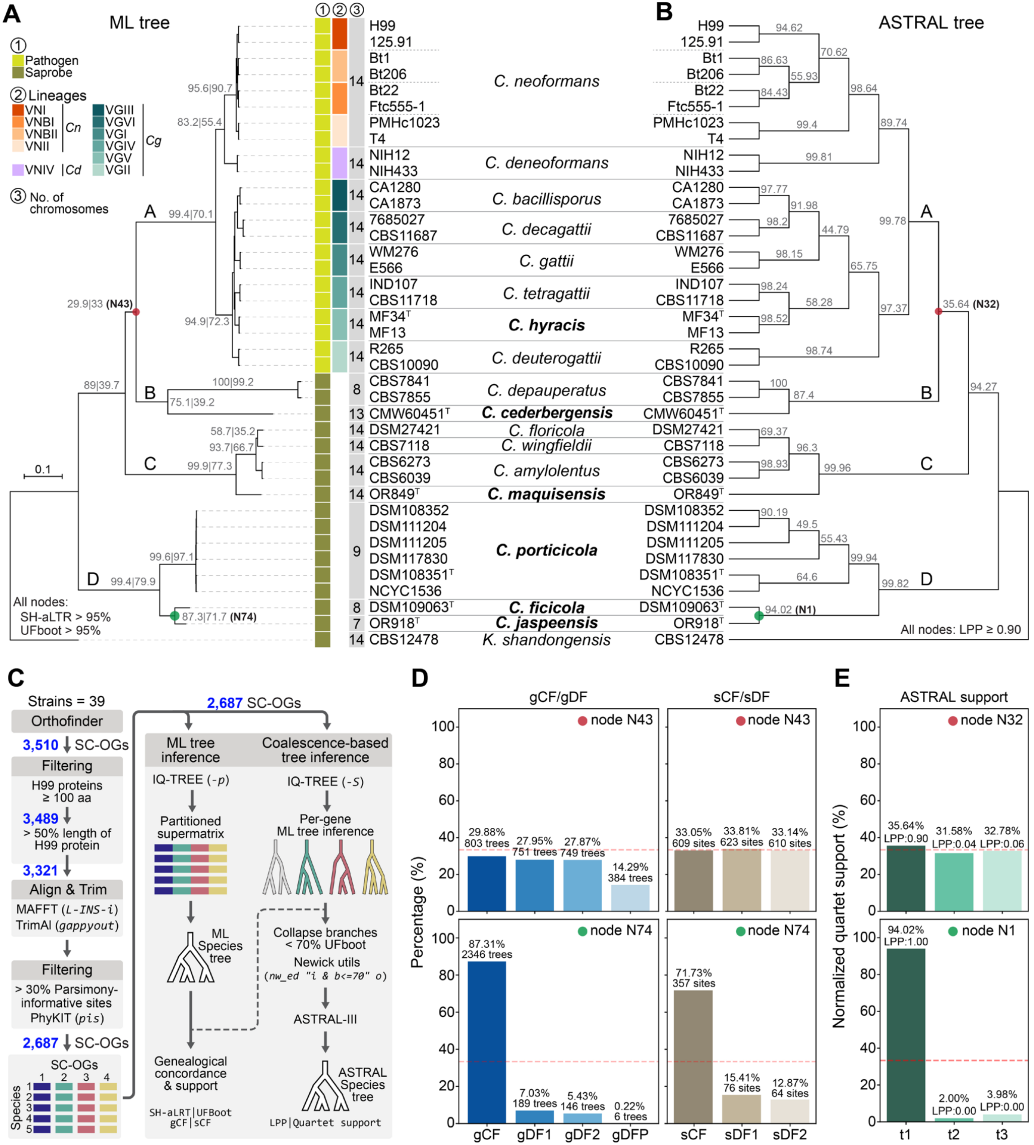
Phylogenomic relationships and species delimitation within *Cryptococcus*. **(A)** Maximum likelihood (ML) phylogeny inferred from a concatenated data matrix comprising protein alignments of 2,687 single-copy orthologs (SC-OGs) across 39 strains, representing 17 *Cryptococcus* species (including six newly proposed species indicated in boldface) and rooted with the outgroup *Kwoniella shandongensis*. Gene and site concordance factors (gCF|sCF) are shown in grey at nodes separating species and major clades. A red circle highlights a conflict-prone node (N43), while a green circle marks an example of a well-supported node (N74). Support values from SH-aLRT and ultrafast bootstrap (UFboot) exceed 95% for all nodes and were omitted for clarity but are available in the corresponding tree file (https://doi.org/10.5281/zenodo.15686462). Branch lengths are shown as the number of substitutions per site (scale bar). Major clades are labeled as partitions A–D. For reference, lineages designations are provided for *C. neoformans* (*Cn*: VNI, VNBI, VNBII, VNII), *C. deneoformans* (*Cd*: VNIV) and *C. gattii* complex (*Cg*: VGI–VGVI), and chromosome numbers are indicated for each species. **(B)** Species tree inferred with ASTRAL, which summarizes gene tree topologies under a multispecies coalescent model, using the same set of 2,687 single-copy orthologs (SC-OGs). Colored circles indicate the corresponding nodes highlighted in panel A, with red marking a conflict-prone node (N32) and green marking an example of a well-supported node (N1). Support values in grey represent the quartet support for the main topology (q1). Local posterior probabilities (LPP) are ≥ 0.90 for all nodes and are not shown for simplicity but are available in the corresponding tree file (https://doi.org/10.5281/zenodo.15686462). Major clades are labeled as partitions A–D. **(C)** Overview of the phylogenomic workflow. A total of 3,510 single-copy orthologs (SC-OGs) were initially identified using OrthoFinder across 39 genomes. These were filtered based on alignment length, sequence completeness, and phylogenetic informativeness, resulting in 2,687 high-confidence SC-OGs. Protein alignments were used to infer individual gene trees and a concatenated supermatrix. The resulting datasets were analyzed with maximum likelihood (ML) methods (panel A) and the coalescent-based ASTRAL approach (panel B). **(D)** Gene and site concordance at two representative nodes: N43 (conflict-prone) and N74 (well-supported). Bar plots on the left show gene concordance factors (gCF) and gene discordance factors (gDF1, gDF2, and gDFP), representing the proportion of gene trees that support the main topology (gCF, dark blue), each of the two alternative topologies (gDF1 and gDF2, light blue), or that display conflicting relationships due to paraphyly of one or more clades (gDFP, pale blue). The corresponding site concordance (sCF) and site discordance (sDF1 and sDF2) values are shown on the right, based on site-wise likelihood support for each topology. The red dashed line at 33.3% indicates the expected support under a hard polytomy, where all three resolutions are equally likely due to lack of phylogenetic signal. **(E)** ASTRAL quartet support for the same nodes shown in panel D: N32 (conflict-prone) and N1 (well-supported). Bar plots show the normalized frequencies of the three possible quartet topologies around each node, based on gene tree topologies. The main topology is shown in dark green, with alternatives in lighter shades. Local posterior probabilities (LPP) for each topology are indicated above the bars. The red dashed line (33.3%) reflects the expected distribution under a hard polytomy.

We previously analyzed the genomes of 17 *Cryptococcus* species, among which six represent novel lineages that remained undescribed and unnamed [25, 27]. These candidate species are distributed across multiple major clades of the genus (defined as clades A to D). To further resolve their phylogenetic placement and support formal species recognition, we reconstructed a robust species phylogeny for *Cryptococcus* based on 2,687 single-copy orthologs (SC-OGs) identified across 39 representative strains (Fig 1A). Where possible, two strains per species were included to assess monophyly and capture intraspecific heterogeneity. For *C. neoformans*, we further incorporated two representatives from each of the major lineages (VNI, VNBI, VNBII, VNII) to reflect known within-species diversity. Sampling within clade D was also expanded by sequencing, assembling, and annotating the genomes of three additional strains. Two of these (DSM108352 and DSM111204) were sequenced with Oxford Nanopore technology, polished with Illumina reads, and assembled to telomere-to-telomere completeness. A third strain (DSM111205) was sequenced with Illumina only, resulting in a high-quality draft assembly. In addition, the publicly available genome assembly of DSM117830 (GCA_024271845.1) was retrieved from GenBank and annotated in this study.

To infer the species phylogeny, we applied a stringent ortholog selection pipeline (Fig 1C), starting with 3,510 SC-OGs and filtering based on minimum protein length, coverage relative to the *C. neoformans* H99 reference proteome, and parsimony informativeness. A final set of 2,687 high-confidence SC-OGs was retained for phylogenetic inference. Both Maximum Likelihood (ML) and coalescent-based analyses (ASTRAL) produced largely congruent topologies with strong support across most nodes (Figs 1A–B). These analyses robustly recovered the major lineages of *Cryptococcus* pathogens (clade A)—*C. neoformans*, *C. deneoformans*, and the various *C. gattii* species (sensu lato)—and clearly separated nine saprobic species into three distinct, deeply diverging clades. Several of the newly proposed taxa formed long branches or well-supported monophyletic groups distinct from previously known species, supporting their recognition as separate evolutionary lineages. Clade B includes *C. depauperatus* and strain CMW60451, representing a new species we designate *Cryptococcus cederbergensis* sp. nov., isolated from a bark beetle (*Lanurgus* sp.) in the Cederberg Mountains of South Africa. Clade C comprises *C. floricola*, *C. wingfieldii*, and *C. amylolentus*, along with a divergent lineage represented by a single strain, now named *Cryptococcus maquisensis* sp. nov., previously isolated from soil in Parque Natural da Arrábida, Portugal [28]. Clade D, sister to all other *Cryptococcus* lineages, contains three well-resolved branches, each representing a new species. One of these includes six closely related strains that form a strongly supported monophyletic lineage, designated *Cryptococcus porticicola* sp. nov., named for its occurrence in bark beetle galleries, from which several strains were isolated in Russia and Sweden. An additional strain was isolated from the gut of a bark beetle larva collected in the Czech Republic. The other two branches represent *Cryptococcus ficicola* sp. nov., isolated from a fig in Umbria, Italy, and *Cryptococcus jaspeensis* sp. nov., isolated from soil at Pedreira do Jaspe in Parque Natural da Arrábida, Portugal, with the species name referencing its topographic origin. Additional strain details are provided in S1 Table and in their respective descriptions in the Taxonomy section.

Among the four major clades, the placement of clades B and C relative to clade A was consistent between phylogenetic methods, but this relationship (node N43 in Fig 1A; node N32 in Fig 1B) exhibited comparatively lower gene and site concordance factors (gCF = 29.9%, sCF = 33.05%; Fig 1D), and lower quartet support (35.64%; Fig 1E), indicating persistent conflict at this deep node despite topological agreement. While not a strict cutoff, a threshold near 33% is often applied in both frameworks to assess phylogenetic signal. This value reflects the theoretical expectation that, among the three possible unrooted topologies for a given internode, support below this level suggests that alternative relationships are more frequently favored than the main topology.

To assess whether similar patterns of conflict extended to shallower nodes within clade A, we examined a pruned view of the ML and ASTRAL trees, restricted to species in the *Cryptococcus* pathogenic clade (S1A–B Figs). The overall topology was identical between methods, but several internal nodes (N47, N52, N61 in the ML tree) showed relatively low concordance. At these nodes, the main topology was most frequently supported by gene trees (gCF = 26.6–32.7%), yet a substantial proportion of trees were discordant or paraphyletic (gDFP ~46%) (S1C Fig). Likewise, site concordance factors (sCF) only modestly favored the main topology. ASTRAL quartet support for the corresponding nodes (N28, N23, N16; S1D Fig) consistently identified the primary topology (t1) as best supported, with normalized support ranging from 44.8% to 58.3%, and all nodes having local posterior probabilities (LPP) above 0.9. These results suggest that while gene tree conflict is common at these shallow nodes the dominant signal still supports a stable species tree. Such patterns are frequently associated with incomplete lineage sorting (ILS) and recent speciation events [29, 30].

### Genome-wide divergence metrics support species boundaries in the *Cryptococcus gattii* complex and the formal recognition of *Cryptococcus hyracis* sp. nov.

In addition to the newly identified nonpathogenic species, we formally recognize a previously described but unnamed lineage within the *C. gattii* complex. This lineage, referred to as VGV by Farrer et al. [15], was discovered in the Central Zambezian Miombo Woodlands of Zambia and isolated from tree holes and hyrax middens (stratified fecal and urinary deposits formed at communal latrines). In that study, VGV was shown to be genetically distinct from other *C. gattii* lineages (VGI–VGIV), based on both population genetic analyses and its placement as a monophyletic group in genome-wide phylogenies. Species names for all major molecular types (VGI–VGIV and VGVI) had been formally proposed by Hagen et al. in 2015 [10], establishing a precedent for taxonomic recognition within the complex. Because a subsequent publication expressed caution regarding the stability and interpretation of species boundaries in this group [16], the VGV lineage remained unnamed despite clear evidence of genetic divergence. To maintain consistency with the framework proposed by Hagen et al. [10, 13], to reflect our view that recognizing distinct species within the *C. gattii* complex is advantageous for genomic and ecological clarity, we propose to designate the VGV lineage as *Cryptococcus hyracis* sp. nov., in reference to its frequent association with hyrax-inhabited environments.

In our phylogenetic analyses, *C. hyracis* formed a well-supported clade distinct from its closest relative, *C. tetragattii* (VGIV) (Figs 1A–B and S1A–B). The two species were resolved as sister groups in the ASTRAL tree with moderate quartet support (58.3%, node N23; S1B and S1D Figs), and gene concordance fell just below the 33% threshold for inferring a dominant phylogenetic signal (gCF = 32.7%, node N52; S1A and S1C Figs), again pointing to potential ILS or rapid divergence.

To complement the phylogenetic analyses and further evaluate species boundaries, we calculated genome-wide divergence metrics including average nucleotide identity (ANI), average amino acid identity (AAI), and digital DNA–DNA hybridization (dDDH) across species. These metrics, widely adopted in prokaryotic taxonomy, are increasingly applied to yeast systematics under the taxogenomic framework to quantify genetic relatedness beyond traditional barcode sequences [31]. ANI measures nucleotide-level similarity between homologous genomic regions, with values above ~95–96% typically indicating conspecificity in prokaryotes. Similarly, dDDH, originally developed to approximate wet-lab DNA–DNA hybridization, estimates intergenomic relatedness using BLAST-based comparisons and retains the conventional 70% threshold for species delineation [32]. However, the thresholds developed for prokaryotes are not universally applicable to fungi, and their application in yeast remains underdeveloped. Broader application will likely require integrating additional criteria, such as sexual compatibility and reproductive isolation under the biological species concept. While limited in scope and largely focused on ascomycetous yeasts, initial studies suggest these metrics can be informative when aligned with phylogenetic and phenotypic data [31, 33].

**Fig 2.**
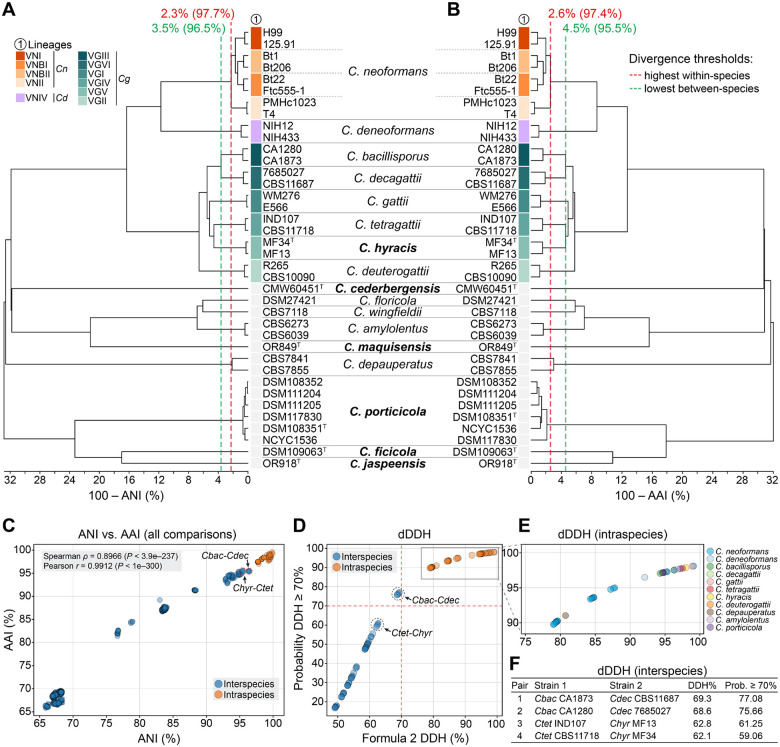
Genomic divergence and species boundaries among *Cryptococcus* species. **(A–B)** UPGMA clustering based on pairwise sequence divergence, calculated as 100 – Average Nucleotide Identity (ANI) in panel A and 100 – Average Amino Acid Identity (AAI) in panel B. Vertical dashed lines indicate divergence thresholds: red marks the highest observed divergence within a species (i.e., the lowest ANI or AAI between *C. neoformans* lineages), and green marks the lowest observed divergence between species (i.e., the highest ANI or AAI between *C. bacillisporus* and *C. decagattii*). **(C)** Scatterplot comparing ANI and AAI values across all pairwise genome comparisons. Intraspecies comparisons are shown in orange and interspecies comparisons in blue. The two interspecies pairs with the highest genomic similarity are highlighted: *C. bacillisporus* (*Cbac*) vs. *C. decagattii* (*Cdec*), and *C. hyracis* (*Chyr*) vs. *C. tetragattii* (*Ctet*). Pearson and Spearman correlation coefficients indicate a strong positive relationship between ANI and AAI values across all comparisons. **(D)** Digital DNA–DNA hybridization (dDDH) estimates for pairwise genome comparisons. The x-axis shows values derived from *Formula 2* of the Genome-to-Genome Distance Calculator (GGDC), which estimates the expected wet-lab DDH percentage based on genome BLAST comparisons. The y-axis shows the probability that the true DDH value exceeds 70%, a widely accepted threshold for species demarcation. Dashed lines indicate the 70% threshold for both axes. Intraspecies comparisons are shown in orange and inter-species comparisons in blue. **(E)** Zoomed-in view on intraspecies dDDH comparisons, grouped by species. **(F)** Table of dDDH values for select interspecies comparisons that approach or exceed the 70% threshold, including the genome pairs with the highest interspecific scores. *C. bacillisporus*–*C. decagattii* comparisons fall just below the 70% cutoff but have high probabilities (>75%) of exceeding it, indicating borderline genomic divergence despite their current recognition as separate species. In contrast, *C. tetragattii*–*C. hyracis* comparisons fall well below both thresholds, supporting their recognition as distinct species.

To contextualize these metrics for species delimitation and ensure their appropriate application to basidiomycete fungi, *C. neoformans* was selected as a benchmark. This well-circumscribed species exhibits a documented sexual cycle and reproductive isolation. Genome comparisons included eight *C. neoformans* strains, with two representatives from each of the major lineages (VNI, VNII, VNBI, and VNBII), providing a reference for intraspecific variation. Although these lineages exhibit substantial population structure and limited recombination between them, they are recognized as a single species [34]. Thus, the lowest similarity observed among these strains provides a conservative upper bound for intraspecific genomic divergence within *Cryptococcus*. The minimum ANI and AAI values observed among *C. neoformans* lineages were 97.7% and 97.4%, respectively, corresponding to divergences of 2.3% and 2.6% (Fig 2A–C and S2 Table). Similarly, to provide a reference point for the lower bound of interspecific divergence, we used the species pair *C. bacillisporus* and *C. decagattii*, which are considered taxonomically distinct despite relatively high genomic similarity. The highest observed ANI and AAI values between strains of these two species were 96.5% and 95.5%, respectively, corresponding to divergences of 3.5% and 4.5%. These values fall below the lowest ANI and AAI observed in any intraspecific comparison in our dataset (Fig 2C and S2 Table), reinforcing the interpretation of *C. bacillisporus* and *C. decagattii* as a species-level boundary case.

Comparatively, pairwise analysis between *C. hyracis* and its sister species *C. tetragattii* yielded ANI values ranging from 95.32% to 95.51% and AAI values from 95.21% to 95.61%, depending on the strain pair. These are lower than the values observed within *C. neoformans* lineages (97.7% ANI and 97.4% AAI) and also lower than those between *C. bacillisporus* and *C. decagattii* (96.3–96.5% ANI; 95.4–95.5% AAI), the most closely related interspecies pair in our dataset (Fig 2A–B and S2 Table). The ANI vs. AAI scatterplot (Fig 2C) reinforced this pattern: *C. hyracis–C. tetragattii* comparisons fell well below the intraspecies cluster and were among the most similar interspecies comparisons, yet still more divergent than *C. bacillisporus–C. decagattii*. The strong correlation between ANI and AAI across all comparisons (Spearman *ρ* = 0.8966, *P* < 3.9e–237; Pearson *r* = 0.9912, *P* < 1e–300) supports the consistency of these metrics in capturing species-level genomic divergence.

Estimates based on dDDH further supported the distinctiveness of *C. hyracis*. Pairwise dDDH values between *C. hyracis* and *C. tetragattii* ranged from 62.1% to 62.8%, with corresponding probabilities of exceeding the 70% species threshold between 59.1% and 61.3% (Fig 2D–F and S2 Table). For comparison, *C. bacillisporus* and *C. decagattii* yielded a dDDH value of approximately 69.3%, just below the conventional cutoff, but with a greater than 75% probability that the true value exceeds the 70% threshold. Nonetheless, these interspecific values remain significantly lower than those observed between *C. neoformans* strains, where dDDH ranges from 79% (probability ≥70% = 89.75%) to 99.1% (probability ≥70% = 98.07) (Fig 2E and S2 Table). This suggests that *C. bacillisporus* and *C. decagattii* lie near the boundary of species-level divergence. In contrast, *C. hyracis* and *C. tetragattii* show a more pronounced level of genomic separation. Together, these results support the recognition of *C. hyracis* as a distinct species, building on previous work by Farrer et al. [15] that first identified it as a divergent lineage within the *C. gattii* complex.

Although all currently available strains of *C. hyracis* are of the *MAT*α mating type and no sexual cycle has been observed thus far, we tested whether *C. hyracis* could engage in mating with strains from other *C. gattii* (sensu lato) species. Our assays showed that *C. hyracis* is capable of forming sexual structures in pairings with *MAT***a** strains of *C. bacillisporus*, *C. decagattii*, *C. tetragattii*, and *C. deuterogattii* (S2 Fig). Notably, strain MF13 consistently exhibited stronger mating responses than MF34, indicating possible strain-level differences in mating competence, consistent with prior observations of intraspecific variation and their placement in distinct subclades [15]. While the fertility and ploidy status of resulting progeny were not assessed here, these preliminary results suggest that *C. hyracis* retains the ability to mate with other species in the complex. A more systematic investigation of post-zygotic outcomes will be needed to assess reproductive compatibility and isolation across the *C. gattii* complex and to test species boundaries under the biological species concept.

### Chromosomal rearrangements and karyotypic diversification in clade A species

**Fig 3.**
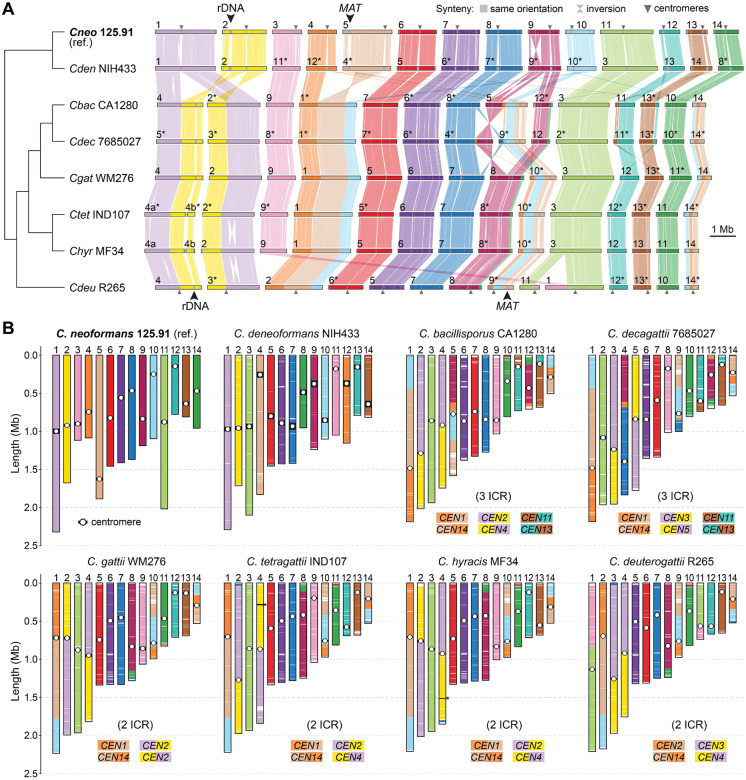
Chromosomal rearrangements among clade A pathogenic *Cryptococcus* species. **(A)** Synteny comparisons between *C. neoformans* strain 125.91 (reference) and representative strains from 7 other clade A species (8 species total). Rearrangements, including large inversions and translocations, were identified using the ntSynt toolkit and visualized with ntSynt-viz. All species maintain 14 chromosomes, but some differences in chromosomal structure are evident. Chromosomes were reordered to maximize collinearity, and those inverted relative to their original assembly orientations are marked with asterisks. The positions of the mating-type locus (*MAT*) and the rRNA locus are also indicated. **(B)** Schematic comparison of chromosome organization across the same strains, highlighting large-scale translocations and centromere locations. Chromosomes are color-coded according to their correspondence with the *C. neoformans* reference. Putative intercentromeric recombination (ICR) events are indicated where synteny breakpoints coincide with centromeres. Asterisks denote chromosomes comprising two contigs interrupted at the rDNA array. Diagrams were generated using a custom Python (available at http://Clinicaltrials.gov) based on pairwise alignments from minimap2.

Structural variation at the chromosomal level provides a complementary axis of genome divergence and may offer important insights into reproductive isolation and the emergence of species boundaries. Although all pathogenic *Cryptococcus* species (clade A) maintain a karyotype of 14 chromosomes (Fig 1A) [27], the extent and pattern of large-scale chromosomal rearrangements across these species have not been systematically compared based on chromosome-level assemblies. To address this, we first performed comparative synteny analyses across clade A species using *C. neoformans* strain 125.91 as a reference. This strain was selected over the more commonly studied H99, which carries a private translocation and therefore does not represent the typical karyotype of the species (S3A–B Figs). Interspecies differences are summarized in Fig 3 with one representative strain per species. S3 Fig presents additional karyotype comparisons among *C. neoformans* and *C. deneoformans* strains, while S4 Fig includes all analyzed strains from the *C. gattii* complex to capture both species- and strain-specific variation.

Our comparisons revealed multiple species- and strain-specific structural differences, including large translocations and inversions. Three translocations shared by *C. bacillisporus* and *C. decagattii* appear to involve centromeric regions of chr. pairs 1–2, 4–5, and 12–13 in *C. neoformans* 125.91 (Figs 3A–B), consistent with intercentromeric recombination (ICR) as a known mechanism of chromosome reshuffling in *Cryptococcus* [35, 36]. Two of these events (1–2 and 4–5) are older, as they are present in all analyzed *C. gattii* (sensu lato) species, suggesting that they originated in their last common ancestor. In contrast, the third event (chr. pair 12–13) is absent in *C. gattii* (sensu stricto), *C. tetragattii*, *C. hyracis*, and *C. deuterogattii*, indicating that it occurred later, in the lineage leading to *C. bacillisporus* and *C. decagattii*.

Additional translocations were identified among *C. gattii* lineages (S4 Fig). For example, chrs. 1 and 11 in *C. deuterogattii* appear to derive from a translocation between corresponding chrs. 3 and 9 of *C. gattii* WM276, which are conserved in related species and likely represent the ancestral state (anc3 and anc9) (Figs 3A and S4B, event a). Two further rearrangements involving anc12–anc13 and anc8–anc10 define the common ancestor of *C. bacillisporus* and *C. decagattii* (S4B Fig, events b and c). In *C. decagattii*, one of the resulting chromosomes (corresponding to extant *C. bacillisporus* chr. 5) underwent a subsequent translocation with anc7, giving rise to extant chrs. 4 and 9 in *C. decagattii* (S4B Fig, event d). We also detected two strain-specific translocations: one in *C. decagattii* CBS11687 (between anc1 and anc11; S4B Fig, event e) and another in *C. tetragattii* CBS11718 (between chrs. anc6 and anc13; S4B Fig, event f). As a result, *C. bacillisporus* differs from *C. decagattii* by one fixed translocation (relative to strain 7685027) or two (relative to strain CBS11687), which further impose postzygotic reproductive barriers.

In contrast, *C. hyracis* strain MF34 and *C. tetragattii* IND107 share a largely collinear karyotype, differing only by a single inversion on chr. 2. Similarly, *C. neoformans* 125.91 and *C. deneoformans* NIH433 differ only by two large inversions (on chrs. 1 and 9; Fig 3), with otherwise conserved chromosomal structures. Although these species pairs show limited karyotypic divergence, their clear sequence-level divergence, particularly in the case of *C. neoformans* and *C. deneoformans*, supports their recognition as distinct species, suggesting that large-scale structural changes may not always accompany speciation.

Together, these results reveal that karyotypic diversification across clade A involves a mixture of lineage-specific rearrangements and ongoing structural changes at the strain level. While fixed chromosomal rearrangements likely reinforce reproductive isolation in some lineages—such as between *C. bacillisporus* and *C. decagattii*—their absence in others, such as *C. hyracis* and *C. tetragattii*, underscores a complex and potentially asynchronous relationship between genome structural variation and species divergence in *Cryptococcus*.

### Intraspecific variation and evidence of recombination in *Cryptococcus porticicola*

Among members of newly described clade D, *Cryptococcus porticicola* is the only species for which multiple strains are currently available and have been sequenced, enabling analyses of intraspecific genome variation, chromosomal structural dynamics, and reproductive potential. To explore these aspects, we first conducted pairwise genome comparisons among the six sequenced strains revealing high overall similarity, with ANI values ranging from 99.29% to 99.94% and dDDH values from 94.6% to 97.2% (Fig 2E and S2 Table), confirming that all isolates belong to the same species despite some geographic separation.

**Fig 4.**
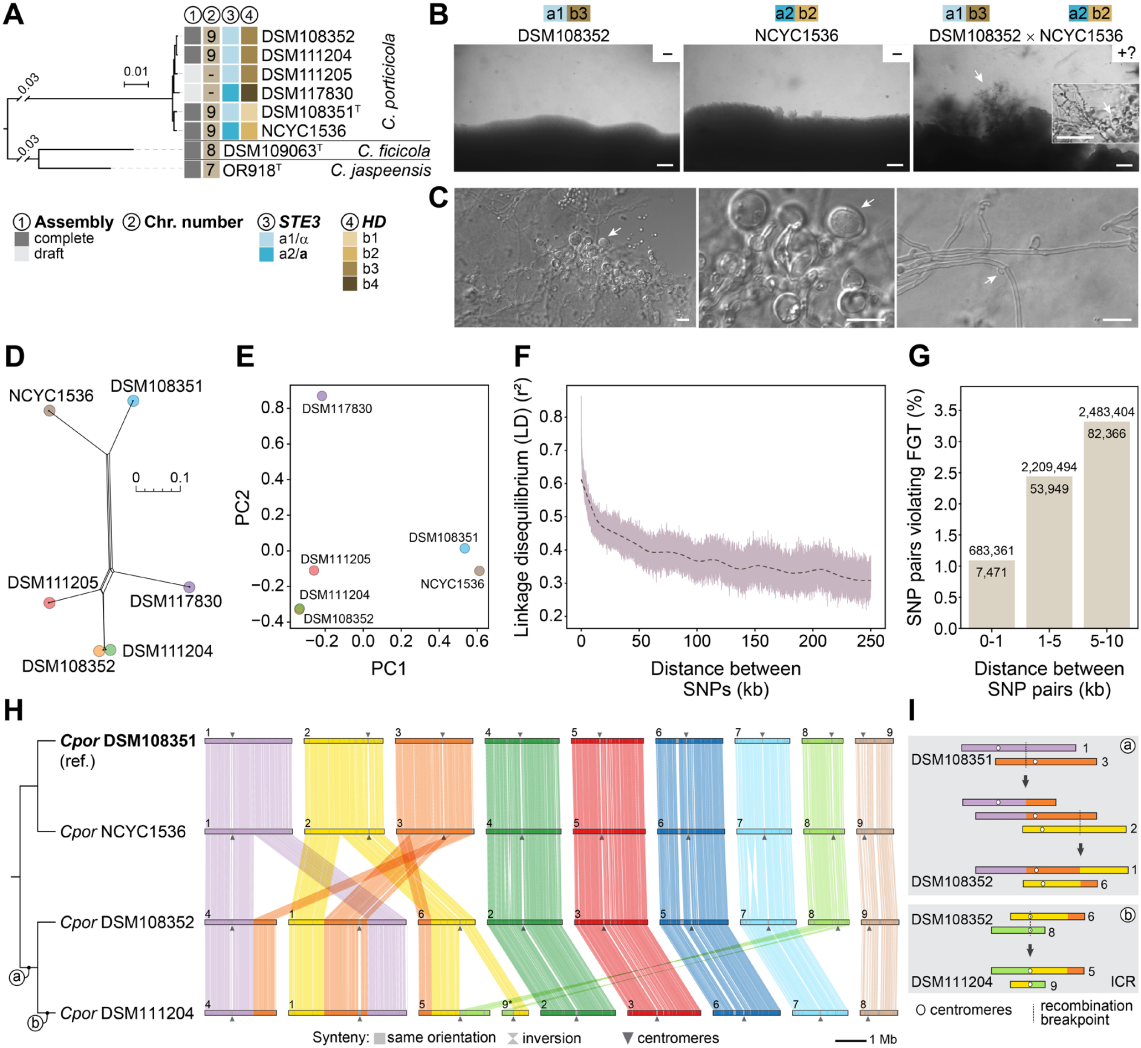
Mating, recombination signatures, and chromosomal structural variation in *Cryptococcus porticicola* **(A)** Maximum likelihood phylogeny of clade D species (pruned from Fig. 1), highlighting *C. porticicola*, *C. ficicola*, and *C. jaspeensis*. Labels indicate genome assembly status (complete or draft), chromosome number inferred from telomere-to-telomere assemblies, mating pheromone receptor allele (*STE3* a1/α or *STE3* a2/a), and *HD* allele identity (b1–b4) inferred from sequence comparisons. **(B)** Mating assay between *C. porticicola* strains DSM108352 (*a1b3*) and NCYC1536 (*a2b2*), showing hyphal formation and putative sexual structures. Solo cultures are shown on the left and center panels; the cross is shown on the right, where hyphal formation is observed embedded in the agar. Basidia-like structures are visible and resemble sexual development in *Kwoniella* species. Assays were performed on V8 medium (pH 5.0) at room temperature in the dark for 1 month. Scale bars: 200 μm (50 μm in the inset). **(C)** Light microscopy images of hyphal and basidia-like structures recovered from the mating plate shown in panel B. The left panel shows hyphae and basidia-like cells (arrows), with a zoomed-in view in the middle panel. The right panel highlights a hyphal segment with an apparently unfused clamp connection (arrow). Scale bars: 10 μm. **(D)** Neighbor-Net phylogenetic network constructed from core SNPs showing signals of conflicting phylogenetic relationships (reticulation), consistent with recombination. **(E)** Principal component analysis (PCA) of core SNP data showing genetic structure among the six strains. The first two principal components explain 46.5% and 21.3% of the total variance, respectively. **(F)** Linkage disequilibrium (LD) decay curve showing mean pairwise r^2^ values (minor allele frequency, MAF ≥ 0.167) as a function of physical distance between SNPs. Dashed line shows lowess-smoothed trend. **(G)** Four-gamete test (FGT) showing increasing proportions of SNP pairs violating the test with physical distance, indicative of recombination. Total SNP pair counts (top numbers) and violating pairs (bottom numbers) are shown for each bin. **(H)** Chromosomal synteny comparisons across *C. porticicola* employing strain DSM108351 as reference. Each block represents a chromosome; ribbons denote orthologous regions as determined by the ntSynt toolkit. Chromosomes were reordered to maximize collinearity, and those inverted relative to their original assembly orientations are marked with asterisks. **(I)** Detailed view of intraspecific chromosomal rearrangements in *C. porticicola*. Centromeres (circles) and synteny breakpoints are shown for each rearrangement. ICR: intercentromeric recombination.

Next, we conducted mating assays between strain pairs predicted to be compatible based on sequence differences at both the pheromone/receptor (*STE3*) and homeodomain (*HD*) mating-type loci (Fig 4A). These two unlinked *MAT* loci were previously characterized and shown to follow a tetrapolar configuration in *C. porticicola* and other clade D species [25]. Among the tested combinations (S5 Fig), only the cross between strains DSM108352 (*a1b3*) and NCYC1536 (*a2b2*) sporadically produced visible hyphae and putative sexual structures embedded in the agar, resembling basidia (Fig 4B–C). These structures emerged only after extended incubation (~1 month) and were not consistently observed across replicates. While their identity could not be definitively confirmed, their morphology is consistent with sexual development observed in related *Kwoniella* species [37–39]. These structures were observed on V8 medium pH 5.0, whereas growth on MS medium led to hyphal formation but without basidial-like clusters (Fig S5D). Notably, some hyphal segments appeared to contain unfused clamp connections (Fig 4C), potentially indicating a monokaryotic mycelium.

To further investigate genetic diversity and potential recombination in *C. porticicola*, we analyzed genome-wide single nucleotide polymorphisms (SNPs) across the six available strains. High-confidence SNPs were identified with Snippy pipeline using DSM108351 as the reference, generating a core genome SNP matrix. A neighbor-net network constructed from this alignment revealed reticulate relationships, suggesting conflicting phylogenetic signals and potential recombination events (Fig 4D). This interpretation is further supported by the Pairwise Homoplasy Index (PHI) test implemented in SplitsTree, which returned a highly significant result (*P* < 1e-6), suggesting that the observed pattern of site incompatibilities is unlikely to arise under a strictly clonal (non-recombining) model. Principal component analysis (PCA) also indicated genetic differentiation among strains, with DSM108352 and NCYC1536—the only cross producing putative sexual structures—showing the greatest separation along PC1, which accounts for the largest proportion of genetic variance (Fig 4E).

To assess patterns of historical recombination more directly, we next examined linkage disequilibrium (LD) and allelic compatibility among SNPs. LD decay analysis, conducted after applying a minor allele frequency (MAF) threshold of 0.167 to reduce noise from rare variants, revealed a clear decline in *r*^2^ values with increasing physical distance between SNPs, consistent with recombination progressively breaking down associations between alleles (Fig 4F). Based on the smoothed decay curve, *r*^2^ dropped to 50% of its initial maximum (LD50) at approximately 13.9 kb. This signal was reinforced by the four-gamete test (FGT), which showed an increasing proportion of SNP pairs violating the test at greater genomic distances. Such violations are expected when recombination occurs, as the presence of all four possible haplotypes between two biallelic SNPs is unlikely to arise without it, although rare recurrent or back mutations (homoplasy) can also contribute. Taken together, and with the caveat of limited sample size, these findings support the hypothesis that recombination occurs in *C. porticicola*, consistent with the morphological features observed in mating assays that resemble, but do not definitively confirm, sexual development.

Finally, to assess whether chromosomal structural variation parallels the sequence-level diversity observed in *C. porticicola*, we compared chromosome-scale assemblies from four strains spanning the phylogenetic breadth of the species (Figs 4A and 4H). All strains possess 9 chromosomes, indicating a conserved karyotype across the species. Synteny comparisons, using DSM108351 as the reference, revealed a largely conserved chromosomal architecture between DSM108351 and NCYC1536, which are more closely related. Nevertheless, several intraspecific rearrangements were identified (Fig 4H). Two reciprocal translocations are shared between DSM108352 and DSM111204, suggesting these events arose in their common ancestor. Additionally, DSM111204 harbors a third, strain-specific rearrangement with centromere-proximal breakpoints, consistent with an intercentromeric recombination (ICR) event (Fig 4I). These results demonstrate that *C. porticicola* harbors detectable chromosomal structural variation among strains, including translocations that may have functional consequences during meiosis. Notably, the rearrangements observed between DSM108352 and NCYC1536 (the only pair producing putative sexual structures) could contribute to postzygotic isolation through segregation defects, potentially explaining the rarity and incompleteness of sexual development. These findings suggest that *C. porticicola* may comprise distinct populations (e.g. geographic or host-related) with established genomic and structural divergence.

### Chromosome number reduction and centromere dynamics in clade D species

**Fig 5.**
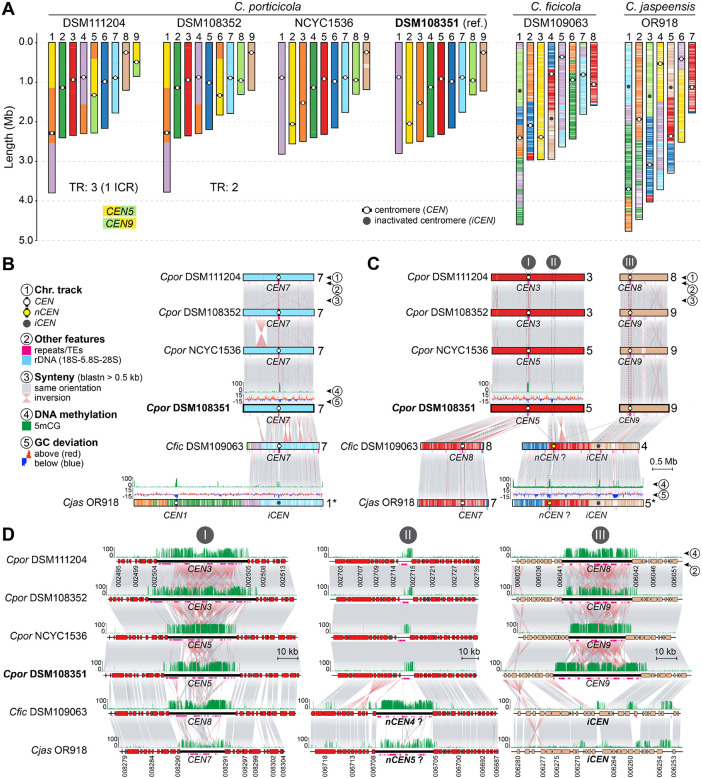
Karyotype evolution and centromere dynamics in clade D *Cryptococcus* species. **(A)** Chromosome-length plots for six telomere-to-telomere assemblies representing *C. porticicola*, *C. ficicola*, and *C. jaspeensis*. Colors indicate synteny blocks, with *C. porticicola* DSM108351 as the reference. Chromosome number differs across species (9, 8, and 7, respectively), and multiple structural changes are observed, including species- and strain-specific translocations (TR). Circles mark predicted centromeres: white for active centromeres (*CEN*) and dark gray for putative inactivated centromeres (*iCEN*). For *C. porticicola*, the total number of translocations (including those consistent with intercentromeric recombination, ICR) is noted below each strain. **(B)** Example of centromere loss in *C. jaspeensis*. A region syntenic to *CEN7* in *C. porticicola* and *C. ficicola* appears inactivated in *C. jaspeensis* OR918 (*iCEN* on chr. 1), but still shows 5mCG methylation, (see S6F Fig for a zoomed-in view of this region). Track descriptions are provided on the key on the left. **(C–D)** Synteny comparisons showing centromere inactivation and possible neocentromere formation in *C. ficicola* and *C. jaspeensis*. In panel C, *CEN5* of *C. porticicola* DSM108351 aligns with *CEN8* in *C. ficicola* and *CEN7* in *C. jaspeensis*, suggesting a shared ancestral origin (zoomed view in panel D, region I). By contrast, *CEN9* in *C. porticicola* appears to have been inactivated (*iCEN*) in both *C. ficicola* and *C. jaspeensis*, coinciding with reduced transposable element content and diminished 5mCG methylation signal, and conservation of flanking genes aside from a minor inversion (panel D, region III). Notably, the current centromere locations in chr. 4 of *C. ficicola* and chr. 5 of *C. jaspeensis* lack orthology with any of *C. porticicola* centromeres, but the corresponding region in *C. porticicola* exhibits low levels of transposons and 5mCG methylation (panel D, region II). This raises the possibility that a neocentromere (*nCEN*) emerged at this shared position in the common ancestor of *C. ficicola* and *C. jaspeensis*, or alternatively, that this region harbored an ancestral centromere that was subsequently lost or inactivated in *C. porticicola*.

The chromosomal variation identified within *C. porticicola* prompted us to investigate broader trends in karyotype evolution across clade D. All three newly described species in this clade exhibit marked reductions in chromosome number relative to *Cryptococcus* species of clades A and C, which maintain the ancestral 14-chromosome karyotype inferred for the genus [27]. Specifically, *C. porticicola* has 9 chromosomes, while *C. ficicola* and *C. jaspeensis* have only 8 and 7 chromosomes, respectively (Fig 5A).

To elucidate the genomic changes underlying these reductions, we compared chromosome-scale assemblies using *C. porticicola* DSM108351 as a reference and mapped centromere positions (defined as long ORF-free regions enriched in LTR retrotransposons and 5-methylcytosine (5mCG) DNA methylation [35, 40–43]) across all available strains of clade D. In *C. jaspeensis*, the region syntenic to *CEN7* of *C. porticicola* and *C. ficicola* lacks hallmark centromeric features and likely no longer functions as a centromere (Figs 5B and S6F). Similarly, both *C. ficicola* and *C. jaspeensis* contain additional inactivated centromeres (*iCENs*) corresponding to *CEN9* and *CEN8* of *C. porticicola*. These *iCENs* retain conserved flanking genes but are depleted of LTR retrotransposons and show only residual 5mCG methylation, consistent with erosion of centromeric identity while preserving local gene order (Figs 5D, panel III, and S6G).

Notably, the number of inactivated centromeres (*iCENs*) in *C. ficicola* (two) and *C. jaspeensis* (three) exceeds the reduction in chromosome number relative to *C. porticicola*, potentially suggesting that a new centromere formed in their common ancestor. Indeed, the chromosomes that formerly carried *CEN9* (chr. 4 in *C. ficicola*, chr. 5 in *C. jaspeensis*) now harbor an apparently active centromere at a novel location (Figs 5C and 5D, panel II). This putative centromere (*nCEN*) is conserved in both *C. ficicola* and *C. jaspeensis* and exhibits hallmark centromeric features, including transposon enrichment and 5mCG methylation. Interestingly, the syntenic region in *C. porticicola* displays spatially restricted 5mCG enrichment and a small number of transposable elements but does not coincide with any predicted centromere. This raises two possible scenarios: either the region was newly co-opted as a neocentromere in the ancestor of *C. ficicola* and *C. jaspeensis*, or it reflects an ancestral centromere that was retained in these species but lost or inactivated in *C. porticicola*. Importantly, gene order is preserved across these chromosomes, suggesting that centromere repositioning, if it occurred, did so without major local rearrangement. Together, these findings suggest that chromosome number reduction in clade D species, potentially contributing to species diversification, is linked to structural rearrangements followed by centromere inactivation, consistent with a broader model supported by observations in *C. depauperatus* [27] and several other fungi, in which centromere identity erodes through the progressive loss or excision of repeat-rich DNA [44–46].

### Variation in rRNA gene organization across *Cryptococcus* species

**Fig 6.**
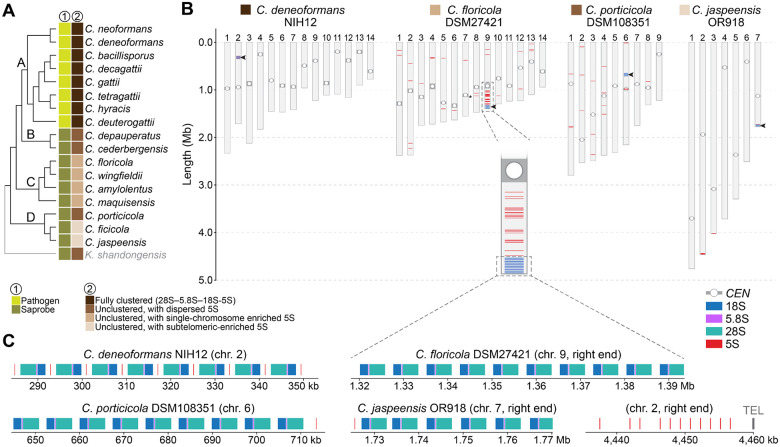
Variation in ribosomal RNA (rRNA) gene organization across *Cryptococcus* species. **(A)** Cladogram of *Cryptococcus* species (adapted from Figure 1), annotated with rRNA gene organization types. All pathogenic species from clade A exhibit a fully clustered rRNA array (28S–5.8S–18S–5S). In contrast, saprobic species display unclustered configurations with lineage-specific patterns: dispersed 5S genes (e.g., clade B), 5S enrichment on a single chromosome (e.g., clade C), or subtelomeric 5S enrichment (e.g., *C. ficicola*, *C. jaspeensis* in clade D). **(B)** Genome-wide distribution of rRNA genes (28S, 5.8S, 18S, 5S) across four representative species: *C. deneoformans* (clade A), *C. floricola* (clade C), *C. porticicola*, and *C. jaspeensis* (both clade D). Arrowheads indicate the position of the core rDNA array, and predicted centromeres are marked with open circles. In clade C, 5S genes are significantly enriched on a single chromosome (e.g., chr. 10 in *C. floricola*, see inset), whereas in clade D species, 5S genes are predominantly subtelomeric (e.g., chr. 2 in *C. jaspeensis*). See S7 Fig for additional species and strains. **(C)** Zoomed views of representative rDNA clusters and subtelomeric 5S regions. TEL, telomere.

In addition to chromosome number changes, variation in ribosomal RNA (rRNA) gene organization represents another axis of genome structural divergence in *Cryptococcus* [27]. To explore this in more detail, we applied a unified pipeline that integrates rRNA gene prediction, clustering detection, and statistical enrichment tests (see Material and Methods) to survey the chromosomal arrangement of 5S, 5.8S, 18S, and 28S rRNA genes across 20 chromosome-level genome assemblies representing all major clades and species (Figs 6 and S7).

Fully clustered rDNA arrays (defined as tandem repeats of the canonical 28S–5.8S–18S–5S gene units) were observed exclusively in pathogenic species from clade A. In contrast, other species exhibited unclustered architectures, with the 5S gene physically separated from the 28S–5.8S–18S unit and exhibiting lineage-specific variation in chromosomal distribution. Among clade C species, the 5S genes remained significantly enriched on a single chromosome (chi-squared test, *P* < 0.0001 for all species; Figs 6B and S7, and **Table 3**), suggesting that despite dispersion, the ancestral chromosomal context of the rDNA array may be partly retained. For instance, in *C. floricola*, over 50% of all 5S genes are located on a single chromosome (standardized residual > +11; S3 Table), which also harbors the canonical rDNA array. By contrast, in other clade C species, the 28S–18S–5.8S array appears relocated to alternate chromosomal ends, consistent with interchromosomal mobility potentially mediated by subtelomeric recombination (S7 Fig).

Clade D species also display unclustered rRNA gene organization but with differing 5S patterns. In *C. porticicola*, 5S genes are distributed relatively evenly across chromosomes (chi-squared test, *P* = 0.87; S3 Table). Conversely, *C. ficicola* and *C. jaspeensis* exhibit significant 5S gene enrichment on specific chromosomes (chi-squared test, *P* = 0.0006 and 0.011, respectively) coupled with a strong subtelomeric bias (Fisher’s exact, *P* < 1.12e^−37^ and *P* < 3.03e^−15^, respectively; S3 Table). In both species, the majority of 5S genes reside near chromosome ends, suggesting relocation to subtelomeric regions, possibly through recombination or transposition. Together, these results indicate that 5S rRNA gene organization varies substantially across *Cryptococcus*, with clustered arrays retained in pathogenic species and more dispersed patterns in nonpathogenic lineages, suggesting lineage-specific shifts in rDNA architecture alongside broader chromosomal evolution.

### Phenotypic differentiation among *Cryptococcus* clades

**Fig 7.**
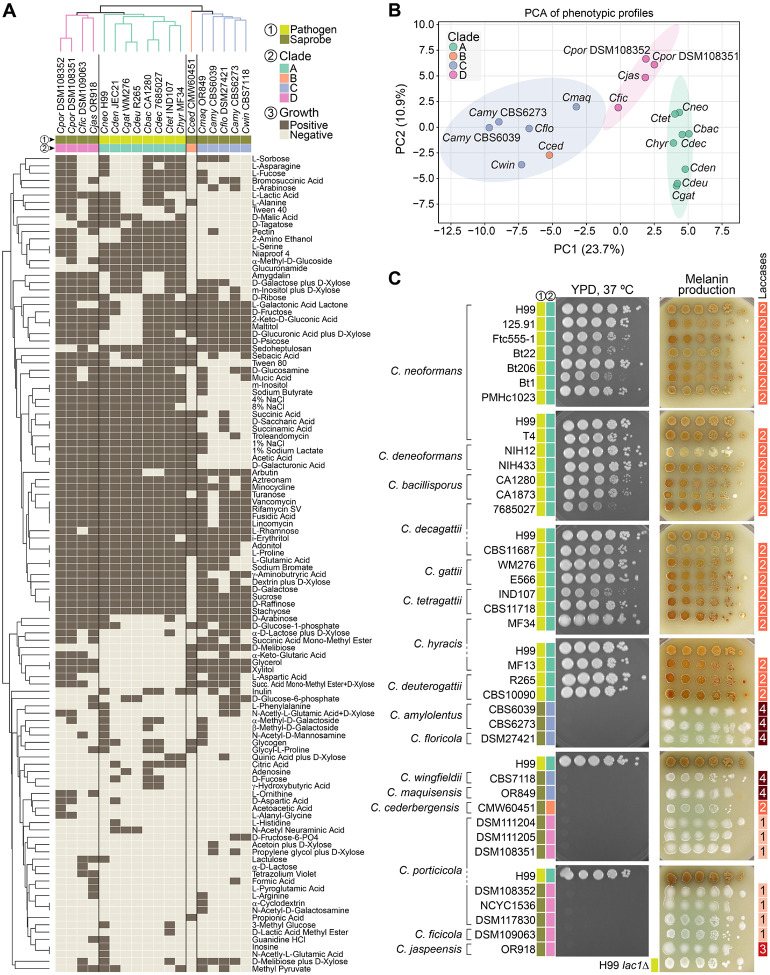
Phenotypic profiling distinguishes *Cryptococcus* clades and pathogenic species. **(A)** Clustermap showing hierarchical clustering of strains (columns) and informative phenotypic tests (rows), based on binary growth/no-growth responses in Biolog assays. Of the 153 substrates tested, 113 showed variable responses across strains and were retained for clustering. Growth was assessed using Biolog YT, FF, and GEN III MicroPlates. Binary values indicate positive growth (brown) or absence of growth (beige). Strains are colored by phylogenetic clade, and clustering was performed using average linkage and Euclidean distance. Most strains cluster according to clade, with metabolic profiles broadly reflecting phylogenetic structure. **(B)** Principal component analysis (PCA) of Biolog growth profiles. The first two components explain 34.6% of the total variance. Ellipses represent 95% confidence intervals for each clade, based on a multivariate normal distribution. Most strains separate by clade, with the exception of *C. cederbergensis* (clade B), which clusters within the clade C ellipse, suggesting phenotypic similarity despite phylogenetic divergence. **(C)** Growth at 37 °C (after 4 days) and melanin production on Bird seed agar (after 5 days at room temperature) across all tested strains. All strains from pathogenic species in clade A exhibited robust thermotolerance and melanin synthesis, whereas strains from saprobic species in clades B, C, and D showed neither trait. The rightmost column indicates the predicted number of laccase genes. Several nonpathogenic strains harbor multiple laccase genes, yet failed to produce melanin, indicating that gene copy number alone does not account for trait expression. See S8C Fig for growth at other temperatures and results on alternative melanin-inducing media (L-DOPA).

To examine whether genomic divergence across the *Cryptococcus* genus is accompanied by phenotypic differentiation, we performed comparative growth and metabolic profiling across 16 of the 17 recognized species. Eighteen strains were tested, including duplicate representatives for *C. porticicola* and *C. amylolentus* (Fig 7 and S4 Table). *C. depauperatus* was excluded due to its markedly slower, filamentous growth, which was incompatible with standardized assay conditions.

We first assessed the metabolic capacity of all strains with Biolog Phenotype MicroArrays, testing growth across 153 substrates. Of these, 113 yielded variable responses across the 18 strains and were retained for comparative analyses (S4 Table). Hierarchical clustering of the binarized profiles revealed clade-level groupings, indicating that species within the same clade tend to share more similar metabolic profiles (Fig 7A). However, the branching order in the dendrogram does not mirror the species phylogeny (Fig 1), suggesting that while combination of metabolic traits can discriminate clades, they may reflect ecological adaptation or functional convergence rather than shared evolutionary history. Principal component analysis (PCA) of the same dataset (Fig 7B) supported broad clade separation along PC1 (23.7%) and PC2 (10.9%). The only exception was *C. cederbergensis* (clade B), which clustered within the 95% confidence ellipse of clade C, suggesting metabolic similarity despite phylogenetic distance. This pattern may, however, change with the discovery and characterization of additional clade B species in the future.

To further explore clade-specific metabolic differentiation, we applied a Random Forest classifier to the binarized Biolog dataset. This analysis identified a ranked set of informative features, with glycerol, glucuronamide, L-serine, xylitol, and D-galacturonic acid among the most discriminating substrates (S8A Fig). Visualization of their average presence across clades revealed clear clade-specific patterns (S8B Fig). For example, glycerol and xylitol were utilized by all clades except clade A; glucuronamide utilization was largely restricted to clade A; and clades B and C showed impaired growth in 4% and 8% NaCl, consistent with heightened osmotic stress sensitivity. Conversely, D-melibiose utilization was restricted to clade C species. These trends suggest that substrate utilization profiles may reflect underlying phylogenetic structure and offer phenotypic markers for clade discrimination. However, because Random Forest identifies correlates of classification rather than causal determinants, further experiments will be needed to validate these candidate traits and assess their diagnostic or taxonomic utility.

In parallel, we evaluated strains from all recognized *Cryptococcus* species (excluding *C. depauperatus*) for two traits broadly associated with pathogenicity: thermotolerance and melanin production. Growth at 37 °C was tested on rich medium, and melanin synthesis was assessed on Niger seed agar and L-DOPA (Fig 7C and S8C). Thermotolerance was sharply restricted to clade A pathogens: all clade A strains grew robustly at 35 °C and 37 °C, while none of the clade B, C, or D strains did. Melanin production showed a similar distribution: all clade A strains produced some level of melanin on both media, whereas all nonpathogenic species lacked detectable production. This pattern was not explained by variation in laccase gene copy number. Laccases are oxidoreductase enzymes that catalyze melanin biosynthesis by oxidizing diphenolic compounds such as L-DOPA [47, 48]. For instance, clade C species such as *C. amylolentus* encode up to four predicted laccase genes but failed to produce melanin under the tested conditions. Together, these results align with prior studies linking thermotolerance and melanin biosynthesis to virulence in *Cryptococcus* and suggest that these traits underlie both ecological adaptation and the evolution of pathogenic potential.

### Taxonomic description of novel *Cryptococcus* species

#### Description of *Cryptococcus hyracis* M.C. Fisher, M. Vanhove, R.A. Farrer, M. David-Palma, M.A. Coelho, and J. Heitman sp. nov.

Etymology: from hyrax (*Dendrohyrax arboreus*), an herbivorous mammal native to Africa and the Middle East; the species name refers to hyrax-associated environments, as several strains, including the type, were isolated from habitats such as tree holes and middens linked to hyrax activity.

After 1 week at 25 °C on PD and GPY streak is white to cream, mucoid with a smooth glistening surface. Margins are smooth and entire. Cells are globose to ovoid 3–8 μm is size, occurring singly or in pairs, and proliferating by multilateral budding. Cells are surrounded by polysaccharide capsules. Pseudohyphae and true hyphae were not observed. Ballistoconidia were not observed. Teleomorph was not observed, as currently available strains are of the same mating type (*MAT*α).

Glucose is not fermented. Positive growth on D-Glucose, D-Galactose, L-Sorbose, D-Xylose, L-Arabinose, L-Rhamnose, Sucrose, Maltose, a,a-Trehalose, Me a-D-Glucoside, Cellobiose, Salicin, Arbutin spliting, Raffinose, Melezitose, Inulin, Starch, Ribitol, Arabinitol, D-Glucitol, D-Mannitol, Galactitol, myo-Inositol, 2-Keto-D-Gluconate, D-Gluconate, D-Glucuronate, D-Galacturonate, Succinate, Citrate, Ethanol, D-glucarate, Galactonic Acid Lactone, Palatinose, L-Malic acid, L-Tartaric acid, Galactaric acid, Gentobiose, and Tween 80. No growth on D-Glucosamine, D-Ribose, D-Arabinose, Melibiose, Lactose, Glycerol, Erythritol, Xylitol, DL-Lactate, Methanol, Propane 1,2 diol, Quinic acid, D-Tartaric acid, and Tween 40.

No growth in the presence of 10% sodium chloride, but on 50% and 60% D-Glucose. Urea hydrolysis and Diazonium Blue B reaction are positive. Starch-like compounds are produced. Maximum growth temperature: 37 °C.

Type: holotype, strain MF34, isolated from a tree hole associated with the Southern tree hyrax (*Dendrohyrax arboreus*), collected on 20 September 2013 in Mutinondo (latitude −12.45, longitude 31.29), Central Zambezian Miombo Woodlands; preserved in a metabolically inactive state in the German Collection of Microorganisms and Cell Cultures (DSMZ), Braunschweig, Germany as DSM 117690.

Molecular characteristics (holotype): nucleotide sequences of ITS and LSU (D1/D2 domains) are available under GenBank accession number PV828371.

#### Description of *Cryptococcus cederbergensis* S. Marincowitz, N.Q. Pham, M. David-Palma, M.A. Coelho, J. Heitman, and M.J. Wingfield, sp. nov

Etymology: refers to the Cederberg Mountains in South Africa, where the sample that yielded the type strain was collected. Formed as a Latinized geographic adjective.

After 1 week at 25 °C on PD and GPY agars, streak is white to cream, mucoid with a smooth glistering surface. On malt extract agar, culture is more mucoid. Margins are smooth and entire. Cells are globose to ovoid 3–8 μm is size, occurring singly or in pairs, and proliferating by multilateral budding. Cells are surrounded by polysaccharide capsules. Pseudohyphae and true hyphae were not observed. Ballistoconidia were not observed. Teleomorph was not observed.

Glucose is not fermented. Positive growth on D-Glucose, D-Galactose, D-Glucosamine, D-Ribose, D-Xylose, L-Arabinose, D-Arabinose, L-Rhamnose, Sucrose, Maltose, a,a-Trehalose, Me a-D-Glucoside, Cellobiose, Salicin, Arbutin spliting, Melibiose, Lactose, Raffinose, Melezitose, Inulin, Starch, Glycerol, Erythritol, Ribitol, Xylitol, Arabinitol, D-Glucitol, D-Mannitol, Galactitol, myo-Inositol, 2-Keto-D-Gluconate, D-Gluconate, D-Glucuronate, Succinate, Citrate, Methanol, Ethanol, D-glucarate, Galactonic Acid Lactone, Palatinose, L-Malic acid, Galactaric acid, Gentobiose, and Tween 80. No growth on L-Sorbose, D-Galacturonate, DL-Lactate, Propane 1,2 diol, Quinic acid, L-Tartaric acid, D-Tartaric acid, and Tween 40.

No growth in the presence of 10% sodium chloride, but not on 50% D-Glucose. Urea hydrolysis and Diazonium Blue B reaction are positive. Starch-like compounds are produced. Maximum growth temperature: 30 °C (weak, slow).

Type: holotype, strain CMW60451 isolated from a bark beetle (*Lanurgus* sp.) infesting twigs of the endangered conifer *Widdringtonia cedarbergensis*, collected on 17 October 2022 in the Cederberg Mountains of South Africa, preserved in the H.G.W.L. Schweickerdt herbarium (PRU). Ex-holotype CMW 60451, preserved in a metabolically inactive state in the culture collection of the Forestry and Agricultural Biotechnology Institute (FABI) at the University of Pretoria, South Africa.

Molecular characteristics (holotype): nucleotide sequences of ITS and LSU (D1/D2 domains) are available under GenBank accession number PV828378.

#### Description of *Cryptococcus maquisensis* M.A. Coelho, M. David-Palma, O. Röhl, J.P. Sampaio, J. Heitman, and A.M. Yurkov, sp. nov.

Etymology: from *maquis*, the French term for dense Mediterranean shrubland, referring to the habitat from which the species was isolated.

After 1 week at 25 °C on PD and GPY agars, streak is white to cream, butyrous to mucoid with a smooth surface. Margins are smooth and entire. Cells are globose to ovoid 3–8 μm is size, occurring singly or in pairs, and proliferating by multilateral budding. Cells are surrounded by polysaccharide capsules. Short pseudohyphae observed in old cultures. No true hyphae were observed. Ballistoconidia were not observed. Teleomorph was not observed.

Glucose is not fermented. Positive growth on D-Glucose, D-Galactose, D-Glucosamine, D-Ribose, D-Xylose, D-Arabinose, L-Rhamnose, Sucrose, Maltose, a,a-Trehalose, Cellobiose, Salicin, Arbutin spliting, Melibiose, Raffinose, Melezitose, Starch, Glycerol, Erythritol, Ribitol, Xylitol, Arabinitol, D-Glucitol, D-Mannitol, Galactitol, myo-Inositol, 2-Keto-D-Gluconate, D-Gluconate, D-Glucuronate, Succinate, Citrate, Ethanol, D-glucarate, Galactonic Acid Lactone, Palatinose, L-Malic acid, L-Tartaric acid, Galactaric acid, and Gentobiose. No growth on L-Sorbose, L-Arabinose, Me a-D-Glucoside, Lactose, Inulin, D-Galacturonate, DL-Lactate, Methanol, Propane 1,2 diol, Quinic acid, D-Tartaric acid, Tween 40, and Tween 80.

Growth in the presence of 10% sodium chloride (weak), and on 50% and 60%D-Glucose. Urea hydrolysis and Diazonium Blue B reaction are positive. Starch-like compounds are produced. Maximum growth temperature: 30 °C.

Type: isotypes of strain OR849 isolated from soil collected in September 2013 in Serra da Arrábida, Setubal, Portugal, preserved in a metabolically inactive state in the German Collection of Microorganisms and Cell Cultures (DSMZ), Braunschweig, Germany (DSM 104548) and the Portuguese Yeast Culture Collection (PYCC), Caparica, Portugal.

Molecular characteristics (holotype): nucleotide sequences of ITS and LSU (D1/D2 domains) are available under GenBank accession number PV828377.

#### Description of *Cryptococcus ficicola* B. Turchetti, M. David-Palma, M.A. Coelho, A.M. Yurkov, and J. Heitman, sp. nov.

Etymology: from Latin *ficus* (fig), referring to the fig fruit, the source of isolation.

After 1 week at 25 °C on PD and GPY agars, streak is white to cream, butyrous to mucoid with a smooth surface. Margins are smooth and entire. Cells are globose to ovoid 3–8 μm is size, occurring singly or in pairs, and proliferating by multilateral budding. Cells are surrounded by polysaccharide capsules. Pseudohyphae and true hyphae were not observed. Ballistoconidia were not observed. Teleomorph was not observed.

Glucose is not fermented. Positive growth on D-Glucose, D-Galactose, D-Ribose, D-Xylose, D-Arabinose, L-Rhamnose, Sucrose, Maltose, a,a-Trehalose, Cellobiose, Salicin, Arbutin spliting, Raffinose, Melezitose, Starch, Glycerol, Erythritol, Ribitol, Xylitol, Arabinitol, D-Glucitol, D-Mannitol, Galactitol, myo-Inositol, 2-Keto-D-Gluconate, D-Gluconate, D-Glucuronate, D-Galacturonate, Succinate, Ethanol, Palatinose, L-Malic acid, Gentobiose, and Tween 80. No growth on L-Sorbose, D-Glucosamine, L-Arabinose, Me a-D-Glucoside, Melibiose, Lactose, Inulin, DL-Lactate, Citrate, Methanol, Propane 1,2 diol, Quinic acid, D-glucarate, Galactonic Acid Lactone, L-Tartaric acid, D-Tartaric acid, Galactaric acid, and Tween 40.

Utilization of nitrogen sources: positive growth on potassium nitrate, sodium nitrite, ethylamine, lysine, and cadaverine.

No growth in the presence of 10% sodium chloride (weak), and on 50% and 60%D-Glucose. Urea hydrolysis and Diazonium Blue B reaction are positive. Starch-like compounds are produced. Maximum growth temperature: 30 °C.

Type: holotype DBVPG 10121 isolated from figs collected in September 2011 in Brufa, Perugia, Italy, preserved in a metabolically inactive state in the Industrial Yeasts Collection DBVPG, Department of Agricultural, Food and Environmental Sciences, University of Perugia, Perugia, Italy. Ex-holotype is preserved in a metabolically inactive state in the German Collection of Microorganisms and Cell Cultures (DSMZ), Braunschweig, Germany as DSM 109063.

Molecular characteristics (holotype): nucleotide sequences of ITS and LSU (D1/D2 domains) are available under GenBank accession number PV828380.

#### Description of *Cryptococcus jaspeensis* M.A. Coelho, M. David-Palma, O. Röhl, J.P. Sampaio, J. Heitman, and A.M. Yurkov, sp. nov.

Etymology: from Alto do Jaspe, a locality in the Arrábida Natural Park (Portugal) where the type strain was isolated. The epithet *jaspeensis* is a Latinized toponym referring to the place of origin.

After 1 week at 25 °C on PD and GPY agars, streak is white to cream, butyrous to mucoid with a smooth surface. Margins are smooth and entire. Cells are globose to ovoid 3–8 μm is size, occurring singly or in pairs, and proliferating by multilateral budding. Cells are surrounded by polysaccharide capsules. Pseudohyphae and true hyphae were not observed. Ballistoconidia were not observed. Teleomorph was not observed.

Glucose is not fermented. Positive growth on D-Glucose, D-Galactose, D-Glucosamine, D-Ribose, D-Xylose, D-Arabinose, L-Rhamnose, Sucrose, Maltose, a,a-Trehalose, Me a-D-Glucoside, Cellobiose, Salicin, Arbutin spliting, Raffinose, Melezitose, Inulin, Starch, Glycerol, Erythritol, Ribitol, Xylitol, Arabinitol, D-Glucitol, D-Mannitol, Galactitol, myo-Inositol, 2-Keto-D-Gluconate, D-Gluconate, D-Glucuronate, D-Galacturonate, Succinate, Ethanol, D-glucarate, Palatinose, L-Malic acid, Galactaric acid, Gentobiose, Tween 40, and Tween 80. No growth on L-Sorbose, L-Arabinose, Melibiose, Lactose, DL-Lactate, Citrate, Methanol, Propane 1,2 diol, Quinic acid, Galactonic Acid Lactone, L-Tartaric acid, and D-Tartaric acid.

Utilization of nitrogen sources: positive growth on potassium nitrate, sodium nitrite, ethylamine, and lysine.

No growth in the presence of 10% sodium chloride (weak), and on 50% and 60%D-Glucose. Urea hydrolysis and Diazonium Blue B reaction are positive. Starch-like compounds are produced. Maximum growth temperature: 25 °C.

Type: isotypes of strain OR918 isolated from soil collected in September 2013 in Serra da Arrábida, Setubal, Portugal, preserved in a metabolically inactive state in the German Collection of Microorganisms and Cell Cultures (DSMZ), Braunschweig, Germany (DSM 104549) and the Portuguese Yeast Culture Collection (PYCC), Caparica, Portugal.

Molecular characteristics (holotype): nucleotide sequences of ITS and LSU (D1/D2 domains) are available under GenBank accession number PV828379.

#### Description of *Cryptococcus porticicola* M.A. Coelho, M. David-Palma, A.V. Kachalkin, T.A. Kuznetsova, M. Kolarik, A.M. Yurkov, and J. Heitman, sp. nov.

Etymology: from Latin *porticus* (meaning gallery, passage, or corridor) referring to the burrows or tunnels made by bark beetles, which is the substrate where the species has been repeatedly found.

After 1 week at 25 °C on PD and GPY agars, streak is white to cream, butyrous to mucoid with a smooth surface. Margins are smooth and entire. Cells are globose to ovoid 3–8 μm is size, occurring singly or in pairs, and proliferating by multilateral budding. Cells are surrounded by polysaccharide capsules. Pseudohyphae and true hyphae were not observed under these conditions. Ballistoconidia were not observed. Under mating conditions on V8 agar (pH 5.0), hyphae and basidia-like structures were sporadically observed after ~1 month of incubation in co-culture with strain NCYC1536. These putative sexual structures appeared embedded in the agar, forming small clusters of globose cells approximately 8–10 μm in diameter. Their morphology was consistent with basidia but their identity could not be definitively confirmed. No sexual structures were observed on MS medium.

Glucose is not fermented. Positive growth on D-Glucose, D-Galactose, L-Sorbose, D-Glucosamine, D-Ribose, D-Xylose, L-Arabinose, D-Arabinose, L-Rhamnose, Sucrose, Maltose, a,a-Trehalose, Me a-D-Glucoside, Cellobiose, Salicin, Arbutin spliting, Raffinose, Melezitose, Starch, Glycerol, Erythritol, Ribitol, Xylitol, Arabinitol, D-Glucitol, D-Mannitol, Galactitol, myo-Inositol, 2-Keto-D-Gluconate, D-Gluconate, D-Glucuronate, D-Galacturonate, Succinate, Ethanol, D-glucarate, Galactonic Acid Lactone, Palatinose, L-Malic acid, Galactaric acid, Gentobiose, Tween 40, and Tween 80. No growth on Melibiose, Lactose, Inulin, DL-Lactate, Citrate, Methanol, Propane 1,2 diol, Quinic acid, L-Tartaric acid, and D-Tartaric acid.

Utilization of nitrogen sources: positive growth on potassium nitrate, sodium nitrite, ethylamine, and lysine.

No growth in the presence of 10% sodium chloride (weak), and on 50% and 60%D-Glucose.

Urea hydrolysis and Diazonium Blue B reaction are positive. Starch-like compounds are produced. Maximum growth temperature: 30 °C.

Type: from gallery of great spruce bark beetle, *Dendroctonus micans* collected in May 2017 in the vicinity of Zvenigorod town, Moscow region, Russia; preserved as two metabolically inactive isotypes in the yeast collection of the Lomonosov Moscow State University (KBP Y-6209) and the All-Russian Collection of Microorganisms (VKM), Pushchino, Russia (VKM Y-3025). Ex-type culture is deposited in the German Collection of Microorganisms and Cell Cultures (DSMZ), Braunschweig, Germany (DSM 108351).

Paratypes: KBP Y-6300 (= DSM 108352 = VKM Y-3027) from the gallery of great spruce bark beetle, *Dendroctonus micans* (in the vicinity of Dmitrov town, Moscow region, Russia), KBP Y-6370 (= DSM 111204 = VKM Y-3517) and KBP Y-6556 (= DSM 111205 = VKM Y-3589) from spruce bark beetle gut, *Ips typographus* (in the vicinities of Nakipelovo and Gzhel villages, Moscow region, Russia), CCUG 11125 (= NCYC 1536 = DSM 117691) from spruce bark beetle gut, *Ips typographus* (Göteborg, Sweden), and CCF 6641 (= DSM 117830) from spruce bark beetle gut, *Ips typographus* (Nižbor, Czechia).

Molecular characteristics (holotype): nucleotide sequences of ITS and LSU (D1/D2 domains) are available under GenBank accession number PV828382.

#### Environmental detection of newly described species and limits of ITS-based detection

To evaluate the potential environmental occurrence of the newly described *Cryptococcus* species, we performed exact-match searches of their ITS1 and ITS2 regions against the GlobalFungi database, which compiles over 80,000 fungal metabarcoding samples from diverse ecosystems [49]. Matches were considered only when query sequences were identical in both length and nucleotide composition. *Cryptococcus porticicola* was detected in nine samples from Europe, New Caledonia, and the United States, primarily from soil and decaying wood. Notably, four samples from northern Europe (Estonia, Finland, and Russia) were from coniferous forests dominated by Norway spruce (*Picea abies*), the typical habitat of *Ips typographus* bark beetles. It was particularly abundant in dead bark samples from the Karelia region [50]. *Cryptococcus maquisensis* was detected in two samples from young *Populus deltoides* xylem collected in Knoxville, Tennessee (USA) [51]. No exact matches were found for *C. cederbergensis*, possibly suggesting a more restricted distribution.

While *C. cederbergensis*, *C. maquisensis*, and *C. porticicola* possess distinct ITS1 and ITS2 sequences, other species exhibit more limited marker resolution. For example, *C. ficicola* and *C. jaspeensis* differ in ITS1 but share an identical ITS2 sequence. ITS1 of *C. ficicola* matched a single air sample from Italy [52], while ITS1 of *C. jaspeensis* returned no matches. The shared ITS2 sequence was recovered from 20 soil samples in Italy, Switzerland [53, 54], and New Caledonia [55]. Similarly, *C. hyracis* shares ITS1 identity with *C. tetragattii*, *C. deuterogattii*, and *C. bacillisporus*, and ITS2 identity with *C. deneoformans*, precluding species-specific identification. It should be noted that the GlobalFungi database is limited in insect-derived samples and lacks bark beetles entirely, providing little insight into insect-associated lineages. A full summary of GlobalFungi matches and ITS1/ITS2 resolution is provided in S5 Table, and an interactive map of matching samples is available as S1 File.

## Discussion

This study expands the known diversity of the genus *Cryptococcus* by formally describing six new species distributed across four distinct clades of the phylogeny. Although initially identified as potential new *Cryptococcus* species through rRNA and later genome sequencing, these taxa had remained undescribed, limiting their integration into broader ecological and evolutionary contexts. Defining species boundaries in fungi, especially among closely related lineages, typically requires a holistic approach that integrates phylogenetic relationships, evidence of reproductive isolation, and other biological traits. However, such data are often difficult to obtain, particularly for non-model organisms with limited experimental tractability or for lineages represented by few isolates. In these cases, genomic data may offer a useful alternative, though interpretation is not without challenges.

As fungal genome resources continue to grow, fungal taxonomy is entering a phylogenomic era in which genome-scale data and quantitative divergence metrics provide a reproducible and evolutionarily grounded basis for species delineation [30, 31]. In this context, taxogenomic approaches anchored in genome-wide comparisons and robust phylogenetic inference are particularly valuable for uncovering hidden diversity and resolving taxonomic uncertainty in yeasts [56, 57]. Still, metrics such as ANI and AAI must be benchmarked against external data that reflect fungal biology, including ecology and reproduction—traits traditionally employed for species circumscription under the phenotypic and biological species concepts [58, 59]. By integrating phylogenomic inference, chromosomal comparisons, divergence metrics, and phenotypic profiling, this study establishes a robust framework for species recognition. While applied here to the genus *Cryptococcus*, this integrative approach can likely be extended to other basidiomycete genera.

In our phylogenomic analyses of *Cryptococcus*, strong overall concordance was observed across inference methods, but residual conflict remained at both deep and shallow nodes. For instance, while the placement of clades B and C relative to clade A was topologically consistent, reduced gene and site concordance and lower quartet support at this deep node point to lingering uncertainty, likely reflecting ancient incomplete lineage sorting (ILS). Within clade A, several internal nodes also exhibited gene tree discordance, likely due to a combination of ILS and recent, rapid speciation. Although historical introgression cannot be entirely ruled out within the *Cryptococcus gattii* complex, available evidence suggests it is rare and limited in scope [15, 60]. Notably, coalescent-based inference with ASTRAL yielded higher resolution across all clade A nodes, with quartet support values exceeding the 33% threshold. These patterns suggest that gene tree discordance, while common in recently diverged fungal lineages, does not necessarily compromise species tree inference, particularly when relationships are reconstructed with coalescent-aware approaches like ASTRAL, which explicitly model the effects of ILS.

Genome-wide similarity metrics such as ANI and ddDDH provide a complementary and quantitative framework for evaluating species boundaries, as demonstrated in prokaryotes. Although fungi lack a universally accepted genomic threshold for species delimitation, a 95% ANI cutoff has been proposed based on limited comparisons across a few selected ascomycetous and basidiomycetous yeasts. However, our comparative analyses support emerging evidence that this threshold may not be suitable and underestimate species-level divergence in some basidiomycete lineages [31, 61]. For example, the lowest ANI and dDDH values observed among *Cryptococcus neoformans* strains (97.7% and 79%, respectively) provide a conservative lower bound for intraspecific genomic similarity, while the divergence between *C. bacillisporus* and *C. decagattii* (96.5% ANI; 69.4% dDDH), two recognized species, establishes a practical lower bound for interspecific separation.

The newly described species *C. hyracis* sp. nov. illustrates the utility of this framework. Phylogenomic analyses resolved *C. hyracis* as sister to *C. tetragattii*, with ANI values ranging from 95.32% to 95.51%, and dDDH values from 62.1% to 62.8%. These values fall below both the intraspecific thresholds observed in *C. neoformans* and even the interspecific values seen between *C. bacillisporus* and *C. decagattii*, providing quantitative support for species-level distinction. Although all currently available *C. hyracis* strains are *MAT*α, precluding intraspecific mating assays, interspecific crosses with *MAT***a** strains from other species produced sexual structures, suggesting the lineage retains sexual competence. Further studies evaluating progeny viability and reproductive compatibility with *C. tetragattii* are needed but are constrained by the current lack of *C. hyracis MAT***a** isolates for control crosses. More broadly, systematic reproductive assessments across the *C. gattii* complex would help refine the interpretive power of genome-based divergence thresholds. When standardized and applied in a clade-specific context, these genomic similarity metrics should provide a scalable and reproducible framework for species delimitation, particularly in lineages where experimental crosses are infeasible. The presence of hybrid strains remains a caveat, underscoring the need to integrate genomic evidence with other lines of data. Together, these findings extend earlier support for the distinctiveness of *C. hyracis* [15] and reinforce the broader utility of empirical, lineage-specific benchmarks for taxonomic resolution in *Cryptococcus* and other fungal taxa.

Chromosomal architecture and genome-wide sequence divergence do not always evolve in concert, and both can independently shape species boundaries. For instance, *C. hyracis* and *C. tetragattii* are resolved as sister species and differ by ~4.5% at the genome level, yet their karyotypes are nearly collinear apart from a single large inversion on chromosome 2, which would only impact fertility in the rare case of crossover within the inverted segment. A similar decoupling of sequence and structural evolution is observed between *C. neoformans* 125.91 and *C. deneoformans* NIH433, which diverge by ~12% but differ structurally by only two large-scale inversions (on chromosomes 1 and 9). This pattern echoes observations in other fungal systems. In *Saccharomyces*, for instance, S. *cerevisiae* and *S. paradoxus* retain collinear genomes but produce sterile hybrids due to anti-recombination effects mediated by mismatch repair [62, 63]. In *Cryptococcus*, deletion of *MSH2* (a key component of the mismatch repair system) enhances hybrid viability between *C. neoformans* and *C. deneoformans*, yet fails to restore normal levels of meiotic recombination, implying the presence of additional post-zygotic barriers [64]. Adding further nuance, some *C. neoformans* strains (e.g., H99, Bt65, Bt81) harbor private rearrangements [36, 65], demonstrating that chromosomal changes can arise independently within species and may not reflect broader lineage evolution. In contrast, *C. bacillisporus* and *C. decagattii*, the most closely related species pair in our dataset (~3.5% divergence), exhibit up to two fixed reciprocal translocations between their sequenced strains, despite high genome-wide similarity. Whether these rearrangements initiated speciation or arose secondarily remains unresolved. However, experimental work in *C. neoformans* shows that targeted centromere breakage can generate novel karyotypes that reduce meiotic viability in the absence of sequence divergence, supporting a causal role for chromosomal changes in reproductive isolation [36]. Although often underappreciated, these findings draw further attention to the role of chromosomal rearrangements in fungal speciation and illustrate the importance of broad within-species sampling to fully capture karyotypic dynamics and evolutionary trajectories.

In addition to the structural rearrangements observed among closely related pathogenic species, our analyses revealed more extensive karyotypic shifts in clade D, including multiple reductions in chromosome number. The three newly described clade D species (*C. porticicola*, *C. ficicola*, and *C. jaspeensis*) harbor fewer chromosomes (9, 8, and 7, respectively) than the ancestral 14-chromosome karyotype inferred for *Cryptococcus* and its sister genus *Kwoniella* [27]. Comparative synteny analyses of centromeric regions, which are typically enriched in transposable elements and 5mCG DNA methylation [35, 40, 41], suggest that chromosome number reductions in clade D were primarily driven by structural rearrangements followed by centromere inactivation. Several centromere-syntenic regions retain conserved flanking gene order but lack TE enrichment and display only residual 5mCG, consistent with inactivated centromeres (*iCENs*). This model aligns with patterns reported in *C. depauperatus* [27] and other fungi such as *Verticillium*, *Malassezia* and *Candida* [44, 46, 66, 67], supporting a broader role for centromere inactivation in karyotype evolution. We also identified an intercentromeric recombination (ICR) event in *C. porticicola* strain DSM111204, associated with a strain-specific chromosomal rearrangement. ICR has been reported in both pathogenic and nonpathogenic *Cryptococcus* species [27, 35, 36], and its detection here extends the known phylogenetic distribution of this mechanism.

In both *C. ficicola* and *C. jaspeensis*, the total number of inferred *iCENs* surpasses the number of chromosomes lost when compared to *C. porticicola*, suggesting that centromere inactivation alone does not fully account for their current karyotypes. This raises the possibility that neocentromere formation may have accompanied or followed inactivation events to maintain chromosome stability. A candidate centromere shared by both species, located on chromosomes 4 and 5, respectively, exhibits hallmark centromeric features, including enrichment for transposable elements and 5mCG DNA methylation. The syntenic region in *C. porticicola* shows only partial enrichment for these features, suggesting that it may represent either a newly co-opted centromere in the ancestor of *C. ficicola* and *C. jaspeensis*, or an ancestral centromere that was subsequently lost or inactivated in *C. porticicola*. If this region indeed functions as a centromere, its architecture contrasts with neocentromeres described in *C. deuterogattii*, which were experimentally induced by targeted deletion of native centromeres and typically form at gene-rich loci lacking repeats, transposon accumulation, and DNA methylation, and are often epigenetically unstable [42, 43]. This distinction may reflect either species-specific differences or contrasts between newly formed neocentromeres and those that have stabilized over extended evolutionary periods. Taken together, our findings suggest that both centromere inactivation and centromere-associated recombination are recurrent processes contributing to genome restructuring and karyotype evolution in *Cryptococcus*, with potential implications for speciation.

Ribosomal RNA (rRNA) genes have long served as core phylogenetic and taxonomic markers in fungal systematics. Mirroring the broader chromosomal and centromeric changes observed across *Cryptococcus*, we found lineage-specific variation in the genomic arrangement of the 5S rRNA gene across *Cryptococcus* species. In clade A pathogens, 5S genes remain physically clustered with the 18S, 5.8S, and 28S components on a single chromosome, a configuration common in fungi [68] and likely maintained by constraints related to nucleolar organization and coordinated rRNA dosage control [69]. In contrast, nonpathogenic species exhibit unlinked and frequently dispersed 5S loci, often enriched in subtelomeric regions as seen in clade D species such as *C. ficicola* and *C. jaspeensis*. While some clade C species retain partial enrichment of 5S genes on the same chromosome as the canonical rDNA array, others show evidence of array relocation, consistent with rDNA mobility. These patterns may reflect relaxed functional constraints or increased genome plasticity in saprobic lineages. Mechanistically, the genomic decoupling of 5S from the other rRNA genes may be facilitated by its independent transcription by RNA polymerase III, in contrast to the polymerase I-transcribed 18S, 5.8S, and 28S genes. This transcriptional distinction has been proposed as a key reason for the physical separation of 5S genes in many eukaryotes [70]. Additionally, retroelement-mediated mobility has been hypothesized as a mechanism for 5S gene dispersal, based on shared internal promoters between 5S rRNA genes and short interspersed nuclear elements (SINEs), and documented interactions with long interspersed nuclear elements (LINE) machinery [70, 71]. The subtelomeric bias observed in some *Cryptococcus* species is also consistent with known hotspots of recombination and TE activity [65]. Together, our results indicate that 5S rRNA gene organization is evolutionarily labile in *Cryptococcus*, likely influenced by its independent transcription and potential for mobilization. These lineage-specific differences raise important questions about the functional consequences of rDNA organization, including possible effects on nucleolar structure, rRNA transcriptional regulation, or genome stability. While these impacts remain unclear, the extent of observed divergence suggests that rRNA gene architecture may represent an underappreciated axis of genome evolution in *Cryptococcus* and highpoints this genus as a promising model for investigating rRNA gene dynamics and genome compartmentalization in fungi.

Beyond structural genome evolution, we also explored the recombination potential of *C. porticicola*, the only newly described nonpathogenic species for which multiple isolates of opposite mating types were available. All six strains were isolated from bark beetle galleries or larval guts, indicating a strong ecological association with conifer-infesting beetles. In mating assays, only one cross (DSM108352 × NCYC1536) produced hyphae and embedded basidia-like structures resembling those of *Kwoniella* rather than the typical aerial basidia of *Cryptococcus*, suggesting a potentially divergent mode of sexual development. However, these structures emerged only sporadically and require further validation. Notably, the two mating strains differ by two chromosomal translocations, which are likely to reduce progeny viability. Despite limited morphological evidence, genomic analyses based on a small number of isolates support ongoing sexual reproduction in *C. porticicola*. Genome-wide SNP patterns revealed reticulate relationships, while linkage disequilibrium (LD) decay and four-gamete test violations were consistent with historical recombination. LD decay analysis showed that *r*^*2*^ values dropped to 50% of their initial maximum at approximately 13.9 kb, which is broadly consistent with values observed in recombining *C. neoformans* subpopulations (e.g., VNI, VNBI, and VNBII), where LD50 ranges from 5 to 7.5 kb when analyzed separately [34]. The four-gamete test further supported this inference, revealing a modest but increasing proportion of SNP pairs that violated the four-gamete condition with distance. Although the sample size remains limited, the convergence of these genomic signals suggests that sexual reproduction is maintained in natural populations of *C. porticicola*, with meiotic processes likely contributing to genetic cohesion, evolutionary flexibility, and ecological persistence. Future work will focus on optimizing mating conditions to enable successful crosses between strains with more collinear karyotypes, as all other combinations tested to date have not yielded evidence of mating. In parallel, recovering additional strains from bark beetle-associated substrates may help identify more sexually compatible pairs. If these approaches prove unsuccessful, or if sporulation remains rare or progeny are technically difficult to isolate, introducing selectable markers into compatible strains could provide an alternative means to detect genetic exchange following co-culture, as demonstrated in other non-model *Cryptococcus* lineages [20].

Complementing our genomic analyses, we evaluated phenotypic variation across the *Cryptococcus* genus to determine whether clade-level divergence is mirrored by metabolic traits. Comparative growth profiling revealed clear clade-level differentiation: hierarchical clustering and principal component analysis (PCA) based on Biolog substrate utilization patterns showed that species within the same clade tend to group together phenotypically, broadly reflecting phylogenetic relationships. Notably, *C. cederbergensis* (clade B) clustered within the clade C phenotypic space, suggesting functional convergence potentially linked to similar environmental conditions. Using a Random Forest classifier, we identified a subset of informative substrates—such as glycerol, xylitol, and D-galacturonic acid—with clade-specific utilization. These findings indicate that metabolic profiles may reflect ecological specialization or historical adaptation. While Biolog assays are sensitive to experimental conditions and may not always replicate physiological responses observed in conventional auxanographic tube-based tests, the consistent clade-level growth responses and clustering observed here supports its utility for comparative phenotyping and points to candidate traits for future genomic dissection. Recent studies in Saccharomycotina yeasts have shown that ecological transitions often coincide with extensive remodeling of metabolic gene repertoires [72, 73]. Our phenotype-first approach in *Cryptococcus* provides a framework to identify traits that may reflect lineage-specific ecological strategies and historical adaptation, although direct links to specific gene family expansions or losses remain to be established.

Strikingly, we confirmed that two traits historically associated with virulence in *Cryptococcus*—thermotolerance and melanin production—are restricted to clade A pathogens in our dataset. None of the nonpathogenic species examined displayed these traits under the tested conditions. Yet, in the case of melanin production, they retain a full complement of biosynthetic genes [27], including multiple laccase homologs; several even harbor greater copy numbers than their pathogenic counterparts. In *C. neoformans*, melanin synthesis is largely driven by the cell wall–associated laccase *LAC1*, which plays a central role in oxidative stress resistance and survival within macrophages [74, 75], while its paralog *LAC2* can partially compensate under certain conditions [76]. The retention and potential redundancy of these laccase genes in nonpathogenic lineages suggest an ancestral, potentially preadaptive function shaped by environmental pressures, in line with models where virulence-associated traits arise from exapted ecological adaptations [77]. With respect to thermotolerance, its strict restriction to clade A pathogens reinforces its status as a critical prerequisite infection and survival in mammalian hosts. Stressors encountered during infection, such as elevated temperature, have been shown to trigger transposable element (TE) mobilization in both *C. deneoformans* and *C. neoformans* resulting in hypermutator-like phenotypes and accelerated antifungal resistance [78–80]. Although none of the nonpathogenic species analyzed here can grow at 37 °C, several retain high TE burdens [27], raising the possibility that stress-induced genomic plasticity may also occur in environmental contexts and could, under the right conditions, prime lineages for future adaptation. Investigating whether similar mutational dynamics operate in these lineages could reveal latent evolutionary capacity for pathogenicity. Further work integrating pan-genomic and pan-phenomic analyses, ecological data, and broader taxon sampling will be key to resolving how such traits shape adaptation and diversification across the *Cryptococcus* genus.

The ecological breadth of *Cryptococcus* is now more apparent with the recovery of newly described species from bark beetle galleries, fruit, and soil across diverse geographic regions. Although distinct from the arboreal hollows, avian guano, and forest debris typically associated with *C. neoformans* and *C. gattii* senso lato, these substrates also represent organic-rich, microbially dynamic environments that may support long-term persistence and saprobic growth. These findings demonstrate the importance of environmental sampling in revealing hidden diversity and advancing our understanding of the ecological and evolutionary processes shaping the genus. They also suggest that complex environmental niches may serve as incubators for traits involved in host adaptation and pathogenicity. In this context, the formal recognition of phylogenetically distinct lineages is not taxonomic excess but a necessary step toward cataloging fungal diversity, linking genotypes to phenotypes. While our integrated approach convincingly resolved species boundaries, it also revealed a key challenge for future surveys: ribosomal markers such as ITS and LSU lack the resolution to distinguish closely related *Cryptococcus* species, limiting species-level identification in short-read metabarcoding datasets. This highlights the need for continued development of genome-informed frameworks to enable consistent identification of novel species and strains, which are essential to support long-term study, surveillance, and diagnostic development, especially as some species may emerge as clinically relevant under shifting ecological, climatic, or antifungal selective pressures.

## Materials and methods

### Strains, mating assays, and microscopy

Strains analyzed in this study were routinely cultured on YPD medium (10 g/L yeast extract, 20 g/L Bacto Peptone, 20 g/L dextrose, and 20 g/L agar), unless specified otherwise. *Cryptococcus neoformans*, *C. deneoformans*, and *C. gattii* sensu lato strains were incubated at 30 °C, while other *Cryptococcus* species were maintained at room temperature (20–25 °C). A complete list of strains and associated metadata is provided in S1 Table.

Mating assays were performed by mixing equal amounts of cells from each strain on V8 agar media (10 g/L yeast extract, 20 g/L Bacto Peptone, 20 g/L dextrose, 20 g/L agar; pH 5.0) or Murashige and Skoog (MS) agar media (4.328 g/L MS mix, 16 g/L agar, 1X vitamin mix, pH to 5.8) and incubated in the dark at room temperature (conditions known to induce mating in *C. neoformans*) for up to one month [81]. In the case of *C. porticicola*, incubation was extended due to delayed and sporadic hyphal development, which was first observed around the one-month mark. Plates were regularly monitored microscopically for the development of mating structures. Solo cultures of each strain were also evaluated on V8 pH 5 and/or MS without a compatible partner. Assays were repeated three independent times to ensure consistent phenotypic observations.

To assess hyphal growth, basidia, and spore formation, the edges of yeast colonies and mating patches were examined microscopically and photographed. Specifically for *C. porticicola*, putative sexual structures embedded within the mating media at the patch margins were imaged by excising small agar blocks and gently compressing them between a microscope slide and coverslip. Light microscopy was performed using a Zeiss Axio Scope.A1 microscope equipped with an Axiocam Color camera and ZEN Lite V3.4 software. Samples of sporulating colonies were prepared for scanning electron microscopy (SEM) as previously described [82] and imaged using the Apreo S SEM with an EDS detector (ThermoFisher, USA) at the Shared Materials Instrumentation Facility at Duke University.

### Genomic DNA extraction

High-molecular-weight (HMW) genomic DNA was isolated using a cetyltrimethylammonium bromide (CTAB)-based extraction method as previously described [36], with careful handling to reduce mechanical shearing. DNA purity was assessed with a NanoDrop spectrophotometer (Thermo) by measuring A260/A280 and A260/A230 ratios. DNA integrity and fragment length were evaluated by clamped homogeneous electric field (CHEF) electrophoresis [27]. For Illumina short-read whole-genome sequencing, genomic DNA was extracted using a phenol:chloroform-based procedure [27]. Final concentrations were quantified using the Qubit dsDNA Assay Kit (Invitrogen) on a Qubit fluorometer.

### Genome sequencing

Whole-genome sequencing was performed with Oxford Nanopore and Illumina technologies. Illumina sequencing for strains DSM111204, DSM111205 and DSM108352 was conducted at the Duke University Sequencing and Genomic Technologies (DUSGT) Core. Libraries were prepared with the Kapa HyperPlus kit and sequenced as paired-end 2 × 150 bp reads on Illumina Novaseq. Nanopore sequencing for strains DSM111204 and DSM108352 was performed in-house. Samples were barcoded using the SQK-LSK109 ligation sequencing kit in combination with EXP-NBD104 native barcoding kits. Library preparation followed the manufacturer’s protocols, and sequencing was carried out on R9.4.1 flow cells (FLO-MIN106) for 48–72 hours under default voltage on a MinION Mk1B device. The corresponding version of MinION software available at the time of each run was applied. Additional details on sequencing platforms, basecalling, and demultiplexing for each sample are provided in S6 Table.

### Genome assembly, gene prediction, and functional annotation

Complete nuclear genomes of *C. porticicola* strains DSM111204 and DSM108352 were assembled using Canu [83] with Nanopore long-read data and polished with both Nanopore and Illumina reads, following a previously established workflow [25]. This process included iterative error correction, removal of mitochondrial and rDNA-only contigs, assessment of assembly completeness and telomeric regions, and manual inspection of read coverage using IGV. The draft genome assembly of *C. porticicola* strain DSM111205 was generated using SPAdes v3.15.3 [84] with default parameters. All assemblies and corresponding raw sequencing data have been deposited in DDBJ/EMBL/GenBank under the BioProject numbers listed in S6 Table. Gene models were predicted on repeat-masked assemblies using Funannotate v1.8.9 (https://github.com/nextgenusfs/funannotate), as previously described [25]. Manual inspection and curation were limited to the mating-type loci, including the annotation of short mating pheromone precursor genes. Functional annotation was performed using the Funannotate “*annotate*” module and incorporated PFAM and InterPro domains, Gene Ontology (GO) terms, COGs, CAZymes, fungal transcription factors, MEROPS proteases, secreted proteins, secondary metabolites, and BUSCO groups, as previously detailed [25].

### Ortholog identification, phylogenetic analyses, and topological support

To construct the phylogenomic data matrix, single-copy orthologs (SC-OGs) were identified across all *Cryptococcus* species and strains, along with the outgroup *Kwoniella shandongensis* (GCA_008629635.2), using OrthoFinder v3.0.1b1, as previously outlined [25]. A total of 3,510 SC-OGs shared among all species were identified. To ensure quality and comparability, only proteins from the *C. neoformans* H99 reference strain that were ≥100 amino acids in length and whose orthologs exceeded 50% of the H99 protein length were retained. These filtering steps yielded 3,321 SC-OGs, which were individually aligned with MAFFT (L-INS-i mode) [85] and trimmed with TrimAl (gappyout) [86]. To enrich phylogenetic signal, only alignments with >30% parsimony-informative sites were retained using PhyKIT (*pis*) [87], resulting in a final dataset of 2,687 SC-OGs. Maximum likelihood (ML) and coalescence-based species trees were inferred from this dataset. For ML inference, SC-OG alignments were concatenated into a partitioned supermatrix (39 taxa, 2,687 partitions, and 1,625,459 sites) and analyzed with IQ-TREE using the “-*p*” option [88]. For coalescence-based inference, individual gene trees were inferred with IQ-TREE using the “-*S*” option. Branches with <70% UFBoot support were collapsed using Newick utilities (*nw_ed “i & b* <*= 70” o*), and a species tree was then estimated with ASTRAL v5.7.8. Branch support and genealogical concordance were assessed using SH-aLRT, UFBoot, gene concordance factors (gCF), and site concordance factors (sCF) for ML trees, and local posterior probabilities (LPP) and quartet support for the ASTRAL tree. Phylogenetic trees were visualized and annotated with iTOL v7. Detailed scripts containing software versions, and model parameters used in these analyses, including visualization of gCF/sCF and ASTRAL support, are accessible at https://doi.org/10.5281/zenodo.15686462.

### ANI, AAI and dDDH

Pairwise average nucleotide identity (ANI) values were computed using OrthoANIu [89]. Custom Python scripts were used to parse and reformat the resulting matrix, convert ANI values to distance scores (100 – ANI), and perform average linkage clustering to generate dendrograms, and Newick-format trees visualized with iTOL v7. Minimum, maximum, and mean ANI values between species or clade groupings were also calculated using a metadata file mapping strains to their respective groups. Average amino acid identity (AAI) was computed using EzAAI v1.2.3 [90] with the DIAMOND algorithm and thresholds of 0.4 identity and 0.5 coverage. Pairwise AAI values were parsed using Python scripts and similarly used for clustering and for computing summary statistics across species or clades. Correlation analysis between pairwise ANI and AAI values was assessed using a custom Python script and by computing Pearson and Spearman correlation coefficients. Pairwise digital DNA–DNA hybridization (dDDH) values were estimated through the Genome-to-Genome Distance Calculator (GGDC 3.0; https://ggdc.dsmz.de/ggdc.php#), using Formula 2, which is recommended for incomplete genome assemblies [32]. Due to the requirement for online submission, representative intra- and interspecies genome comparisons were selected and submitted manually (S2 Table). The output files were parsed with a custom Python script, which extracted Formula 2 dDDH values and the associated probability of exceeding the 70% species threshold. Based on these values, comparisons were categorized as intra- or interspecies, and further classified into confidence levels. This analysis was used to assess congruence with ANI and AAI-based classifications and evaluate species boundaries among closely related lineages. Detailed scripts containing software versions and parameters used in these analyses are available at https://doi.org/10.5281/zenodo.15686462.

### Synteny analyses and centromere identification

Synteny relationships were assessed using three complementary approaches. First, for full karyotype comparisons across selected species, we applied the ntSynt toolkit [91] to identify both strain- and species-specific chromosomal rearrangements (Figs 3A, 4H, S3A, and S4A). For clade A comparisons, ntSynt was run with the following parameters: -*d 5*, -*k 21*, -*w 200*, --*block_size 5000*, --*merge 20000*, --*indel 5000, and* --*w_rounds 200 100*. For clade D comparisons, the following parameters were used: -*d 20*, -*k 24*, -*w 200*, --*block_size 1000*, --*merge 10000*, --*indel 5000, and* --*w_rounds 200 100*. Resulting synteny blocks were visualized using ntSynt-viz [92], which combines ribbon-style plotting with chromosome painting. A pruned species tree in Newick format was supplied for each comparison to guide genome assembly order and improve visual coherence.

Second, to examine chromosomal structural conservation and intercentromeric translocations in greater detail, we performed nucleotide-level synteny analyses using a custom Python workflow (*0_synteny_karyoplotter.py*). Genome-wide alignments were generated with minimap2 (v2.17-r941; -*x asm20*), and synteny blocks were parsed from the resulting PAF files. Analyses were conducted in reference-based mode, with all assemblies aligned to a single genome: *C. neoformans* strain 125.91 for clade A comparisons (Fig 3B), and *C. porticicola* DSM108351 for clade D (Fig 5A). Centromere coordinates were provided in BED format and overlaid on the genome plots to highlight centromeric regions and infer intercentromeric rearrangements. All visualizations were generated in Python 3 using Biopython, pandas, matplotlib, and numpy, and the final SVG outputs were manually refined in Adobe Illustrator for publication.

Third, linear synteny plots comparing chromosomes and selected genomic regions in clade D species, particularly around centromeres, were generated with EasyFig v2.2.2 [93] using BLASTN for alignment. Centromere positions and genome-wide GC content were determined as previously described [25, 27]. Centromeres were defined as the longest ORF-free regions enriched in centromere-associated LTR retrotransposons and exhibiting elevated 5mCG methylation, with boundaries set by conserved flanking centromeric genes. GC content and 5mCG methylation profiles were visualized in IGV and precisely overlaid at scale with EasyFig-derived synteny plots in Adobe Illustrator. Scripts are available at https://doi.org/10.5281/zenodo.15686462.

### DNA methylation detection

DNA methylation analysis was performed using a Dorado–Modkit workflow to detect 5-methylcytosine at CpG dinucleotides (5mCG) from Oxford Nanopore sequencing data. Raw signal data in POD5 format were basecalled with Dorado v0.5.2 using the combined model *sup@latest,5mCG_5hmCG@latest,6mA@latest* to simultaneously call canonical bases and modifications. Output BAM files were then realigned to the genome assemblies with the Dorado aligner and sorted with SAMtools v1.10. Methylation signals were extracted and quantified using Modkit v0.2.5-rc1 (pileup mode, --*preset traditional*) to generate both pileup and bedGraph outputs. Centromere-associated methylation profiles were visualized in IGV and plotted to scale in Adobe Illustrator for integration with genome structure and synteny plots.

### rDNA analysis

A unified Python-based pipeline was developed for identifying and analyzing the distribution of rRNA genes across *Cryptococcus* genomes. For each genome, rRNA genes were annotated using Barrnap v0.9 (https://github.com/tseemann/barrnap) with the *“*--*kingdom euk”* setting, and coordinates for 5S, 5.8S, 18S, and 28S rRNA genes were extracted from the GFF output. Canonical rDNA clusters were defined as colocalized 28S–5.8S–18S genes on the same strand within a 5-kb window, optionally including an adjacent 5S gene. Per-genome chromosome plots were generated to display scaled chromosomes, centromeres, and rRNA gene positions. To test whether 5S genes were non-randomly distributed across chromosomes, the expected number of 5S genes per contig was estimated by normalizing contig lengths relative to the total genome size, assuming uniform probability of 5S placement. Observed versus expected values were compared using a chi-squared goodness-of-fit test, implemented with scipy.stats.chisquare. Standardized residuals were calculated to identify contigs with over- or under-represented 5S copy numbers, and results were tabulated per genome. Additionally, to assess 5S enrichment in subtelomeric regions, subtelomeric windows were defined as the first and last 2.5% of each contig, corresponding to 50 kb at each end of a 2 Mb chromosome. The midpoint of each 5S gene was used to determine overlap with these regions. A 2×2 contingency table was generated for each genome comparing the number of 5S genes located inside versus outside subtelomeric windows, relative to the corresponding base pair content. Significance was evaluated using a one-sided Fisher’s exact test (scipy.stats.fisher_exact, alternative=“greater”) to test for enrichment. All gene coordinates, cluster counts, statistical results, and graphical outputs were exported as structured TSV and SVG files for each genome. To enhance figure clarity and readability, plots were refined by adjusting color schemes, modifying labels, and manually adding additional features using Adobe Illustrator. Scripts and raw data files are available at https://doi.org/10.5281/zenodo.15686462 and in S3 Table.

### Variant analysis and phylogenetic network in *C. porticicola*

Variant analysis within *C. porticicola*, was performed with Snippy v4.6.0 (https://github.com/tseemann/snippy), mapping reads to the strain DSM108351 reference genome (--*cpus 28*, --*unmapped*, --*mincov 10*, --*minfrac 0.9*). The resulting core SNP alignment (core.aln) served as input for Neighbor-net network reconstruction in SplitsTree v6.4.14. Recombination was assessed with the Pairwise Homoplasy Index (Phi) test, a permutation-based method implemented in SplitsTree that detects recombination by identifying statistically significant incompatibilities among adjacent sites in an alignment. The test compares observed homoplasy to that expected under a model of clonal evolution, with p-values < 0.05 interpreted as evidence for recombination [94].

### Population structure and recombination analyses in *C. porticicola*

To investigate recombination within *C. porticicola*, we developed a series of analysis scripts (with filenames indicated in parentheses and available at https://doi.org/10.5281/zenodo.15686462) beginning with the conversion of the multi-sample VCF generated by Snippy to PLINK binary format using PLINK v1.9 with the --*vcf* and --*allow*-*extra*-*chr* options (*0_convert_vcf_to_plink.sh*). Principal Component Analysis (PCA) was conducted in PLINK (*1_run_pca.sh*), and the resulting eigenvectors were plotted using Python (*2_plot_pca.py*) with pandas, seaborn, and matplotlib. Linkage disequilibrium (LD) decay was assessed using PopLDdecay (*3_run_ld_decay_compare.sh*) with a maximum inter-SNP distance of 50 kb. To reduce the influence of rare alleles on LD estimates, we applied a minor allele frequency (MAF) threshold of 0.167, corresponding to a 1/3 threshold for biallelic variants in haploid genomes (i.e., at least 2 of 6 strains carrying the minor allele). LD decay was also calculated without MAF filtering for comparison. The resulting files were parsed and plotted using Python (*4_plot_ld_decay.py*), applying a centered rolling mean and LOWESS smoothing to generate smoothed *r*^*2*^ curves. LD50 values were estimated as the distance at which smoothed *r*^*2*^ values dropped to 50% of the initial maximum, providing a quantitative summary of LD decay. To further examine signatures of historical recombination, a custom Python implementation of the four-gamete test was applied to all biallelic SNP pairs within 10 kb windows (*5_four_gamete_test.py*). SNP pairs were binned by physical distance (0–1 kb, 1–5 kb, and 5–10 kb), and the proportion of pairs exhibiting all four possible haplotypes, indicating historical recombination or recurrent mutation, was computed for each bin. Results were visualized as a barplot summarizing violation frequencies per bin.

### Biolog phenotypic assays and clustering analyses

Phenotypic MicroArray tests were performed using Biolog Gen III, FF, and YT microplates. Fresh 3–7-day-old cultures grown on PDA were used for plate inoculations. Yeast cell density in sterile water (for YT and FF microplates) and Inoculation Fluid B (for Biolog Gen III microplates) was adjusted to the values recommended by the manufacturer for YT microplates. Microplates were inoculated with 100 μl of cell suspension per well and incubated in the dark at 25 °C. Plates were read immediately after inoculation using a microplate reader (Varioskan LUX Multimode Microplate Reader, Thermo Fisher Scientific) at 490 nm, 590 nm, and 750 nm, following the manufacturer’s instructions. Subsequent readings were taken every 3–7 days for up to 21 days. Plates were shaken before each reading to ensure a more homogeneous suspension.

Biolog microplates are an end-point measurement platform. Tests that showed a positive signal during the period were scored as positive. Tests showing no signal and values close to the negative control were scored as negative. In cases where color development and turbidity did not provide a clear result, tests were evaluated by comparison with carbon sources commonly utilized by the genus *Cryptococcus*, as previously demonstrated by auxanographic tube tests. Phenotypic characterization of cultures followed the methods described in Kurtzman et al. (2011) [95]. For standard species descriptions, auxanographic tests were performed in liquid media using both tubes and microplates. Tube tests were conducted in 2 ml volumes in 5- and 7.5-ml plastic tubes following the method described by Kurtzman et al. (2011) [95]. Microplate tests were performed in 200 μl volumes using the same media composition as the tube tests. Tubes and plates were shaken every 1–2 days. Growth in tubes was evaluated visually, while growth in microplates was measured at 600 nm using the Varioskan LUX microplate reader. Results were scored as positive or weak when there was clear or reduced growth compared to glucose, and as negative when growth was minimal or absent relative to both the negative control and glucose.

Phenotypic data from Biolog substrate utilization assays were analyzed with a custom Python pipeline (*0_biolog_analysis_pipeline.py*). Input consisted of a manually curated Excel file (S4 Table) containing binary growth data across multiple *Cryptococcus* strains. Dimensionality reduction and clustering analyses were performed to explore global patterns of phenotypic similarity. Principal component analysis (PCA) was conducted on the standardized dataset, and the first two components were visualized with 95% confidence ellipses grouped by clade. A binary clustermap was generated using hierarchical clustering based on Euclidean distance and average linkage. To identify traits most informative for clade-level classification, a Random Forest classifier was trained on the binary matrix treating clade membership as the target variable, and the top 15 features ranked by importance were retained. Clade-wise presence frequencies for these phenotypes were then calculated and visualized. Redundant phenotypes, defined as those with identical binary profiles across all strains, were automatically flagged to reduce overrepresentation. All analyses and visualizations were performed using Python with pandas, scikit-learn, seaborn, matplotlib, and related packages.

### Temperature and melanin production assays

For temperature assays, pre-inocula of each strain were grown in 4 ml of YPD media overnight at room temperature. New tubes containing 4 ml YPD media each were inoculated with 50 μL of each of the overnight-grown pre-inocula and incubated at room temperature for 48 hours. Cells were then pelleted, washed with sterile distilled water, and adjusted to an optical density of 2.0 at 600 nm. Serial 10-fold dilutions were prepared from these normalized cultures, and 3 μL of each dilution was spotted onto YPD agar plates. Plates were incubated at 23 °C, 30 °C, 35 °C, and 37 °C, and photographed after 3 days. For melanin production assays, 3 μL of each dilution was also spotted onto two melanin-inducing media: L-DOPA agar (7.6 mM L-asparagine, 5.6 mM glucose, 22 mM KH_2_PO_4_, 1mM of MgSO_4_.7H_2_O, 0.5 mM L-DOPA, 0.3 μM thiamine, 20 nM biotin and 20 g/L of agar) and Bird seed agar (7% bird seed extract, 0.1% glucose, and 20 g/L agar) [82, 96]. These plates were incubated at room temperature in the dark for 5 days before imaging. All assays were performed in three independent biological replicates to ensure reproducibility of phenotypic results.

### Environmental detection via GlobalFungi database

To screen for environmental presence of the newly described *Cryptococcus* species, we performed exact-match searches against the GlobalFungi release 5 database [49], which compiles ITS1 and ITS2 amplicon data from 84,972 samples across 846 studies. Full-length ITS1 and ITS2 regions were extracted from each genome using ITSx [97] implemented in SEED 2 [98]. Unique haplotypes were identified through dereplication in SEED 2, and only sequences identical in both length and nucleotide composition were considered matches. ITS sequences from additional *Cryptococcus* species included in the phylogenetic analysis (Fig 1) were queried for comparison. Full metadata and hit summaries are available in S5 Table.

## Supplementary Material

Supplement 1

Supplement 2

Supplement 3

Supplement 4

Supplement 5

Supplement 6

Supplement 7

Supplement 8
