# Supplementary material for: Genomic and phenotypic insights into the expanding phylogenetic landscape of the Cryptococcus genus
Source: bioRxiv. 2025 Jul 30:2025.06.18.660340. Originally published 2025 Jun 24. Preprint. [Version 2] doi: 10.1101/2025.06.18.660340 (PMC12262581; doi:10.1101/2025.06.18.660340)
Supplement: Supplementary file 1 [file media-1.pdf]

Supplementary Information for

Genomic and phenotypic insights into the expanding phylogenetic landscape of the *Cryptococcus* genus

Marco A. Coelho[1,§,*], Márcia David-Palma[1,§], Aleksey V. Kachalkin[2,3], Miroslav Kolařík[4], Benedetta Turchetti[5], José Paulo Sampaio[6], Michael J. Wingfield[7], Matthew C. Fisher[8], Andrey M. Yurkov[9], and Joseph Heitman[1*]

§These authors contributed equally as first authors.

*Corresponding authors

Emails: marco.dias.coelho@duke.edu and heitm001@duke.edu

This PDF file includes:

S1 to S8 Figs

Legends for S1 to S5 Tables, and S1 File

Other supplementary materials for this manuscript include the following:

S1 to S5 Tables, and S1 File

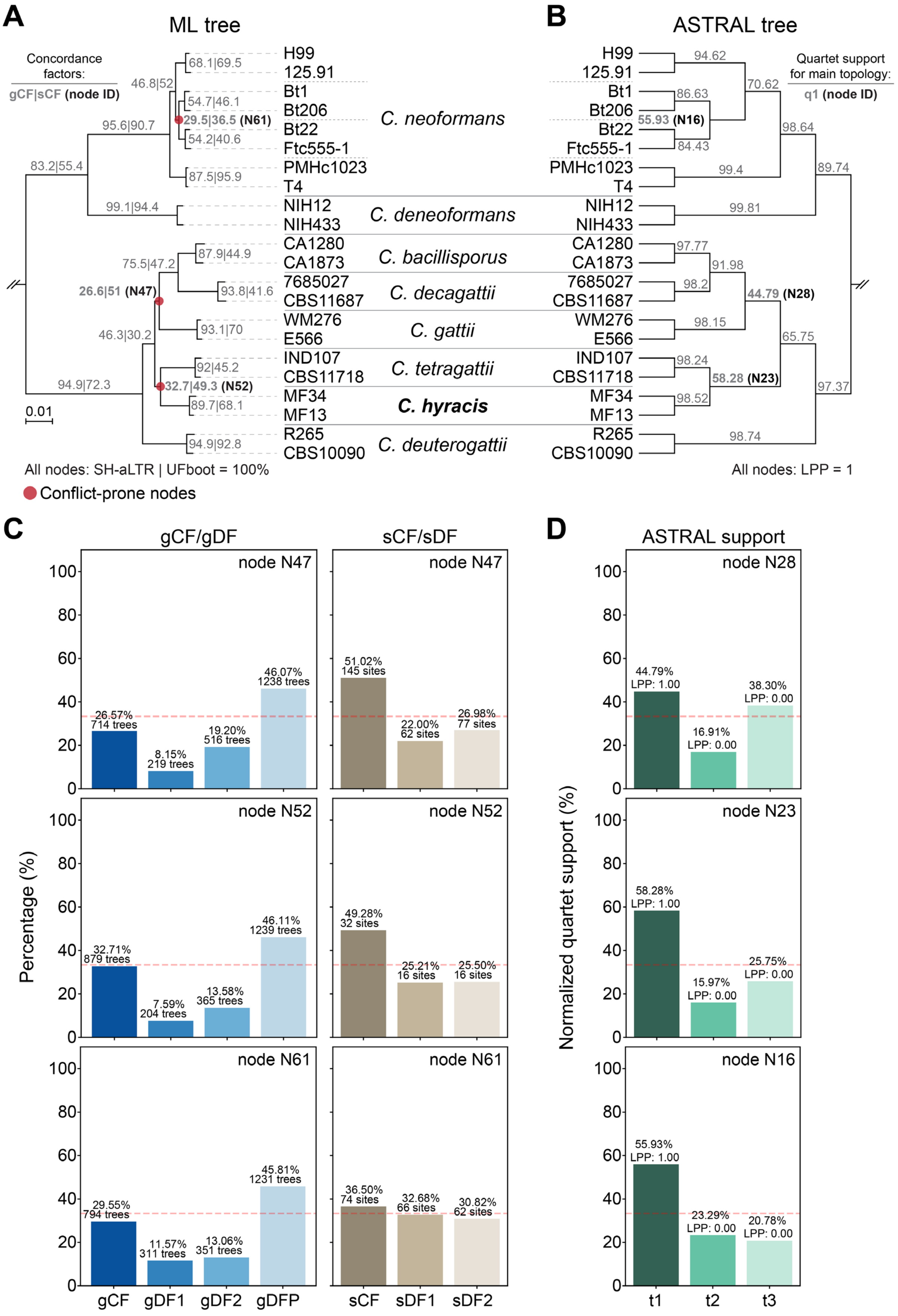

A
ML tree
Concordance factors:
gCF|sCF (node ID)
H99
125.91
Bt1
Bt206
Bt22
Ftc555-1
PMHc1023
T4
C. neoformans
68.1|69.5
46.8|52
54.7|46.1
29.5|36.5 (N61)
54.2|40.6
95.6|90.7
87.5|95.9
83.2|55.4
99.1|94.4
NIH12
NIH433
C. deneoformans
CA1280
CA1873
C. bacillisporus
87.9|44.9
75.5|47.2
7685027
CBS11687
C. decagattii
93.8|41.6
26.6|51 (N47)
WM276
E566
C. gattii
93.1|70
46.3|30.2
IND107
CBS11718
C. tetragattii
92|45.2
32.7|49.3 (N52)
MF34
MF13
C. hyracis
89.7|68.1
94.9|72.3
R265
CBS10090
C. deuterogattii
94.9|92.8
0.01
All nodes: SH-aLTR | UFboot = 100%
Conflict-prone nodes
B
ASTRAL tree
Quartet support for main topology:
q1 (node ID)
94.62
70.62
86.63
55.93 (N16)
84.43
98.64
99.4
89.74
99.81
97.77
91.98
98.2
44.79 (N28)
98.15
65.75
98.24
58.28 (N23)
98.52
97.37
98.74
All nodes: LPP = 1
C
gCF/gDF
sCF/sDF
Percentage (%)
node N47
26.57% 714 trees
8.15% 219 trees
19.20% 516 trees
46.07% 1238 trees
51.02% 145 sites
22.00% 62 sites
26.98% 77 sites
node N52
32.71% 879 trees
7.59% 204 trees
13.58% 365 trees
46.11% 1239 trees
49.28% 32 sites
25.21% 16 sites
25.50% 16 sites
node N61
29.55% 794 trees
11.57% 311 trees
13.06% 351 trees
45.81% 1231 trees
36.50% 74 sites
32.68% 66 sites
30.82% 62 sites
gCF
gDF1
gDF2
gDFP
sCF
sDF1
sDF2
D
ASTRAL support
Normalized quartet support (%)
node N28
44.79% LPP: 1.00
16.91% LPP: 0.00
38.30% LPP: 0.00
node N23
58.28% LPP: 1.00
15.97% LPP: 0.00
25.75% LPP: 0.00
node N16
55.93% LPP: 1.00
23.29% LPP: 0.00
20.78% LPP: 0.00
t1
t2
t3
0
20
40
60
80
100

S1 Fig. Expanded view of phylogenomic support and conflict among clade A *Cryptococcus* species. (A) Pruned maximum likelihood (ML) phylogeny (from Figure 1A), showing only clade A species. The tree was inferred from a concatenated alignment of 2,687 single-copy orthologs (SC-OGs). Conflict-prone nodes are marked in red and labeled by node ID. Concordance factors based on gene trees (gCF) and site patterns (sCF) are shown in grey. All nodes are fully supported by SH-aLRT and ultrafast bootstrap (UFboot = 100%). **(B)** Pruned ASTRAL species tree (from Figure 1B), based on the same SC-OGs. All nodes have local posterior probability (LPP) = 1.0. Quartet support for the main topology is shown in grey. Nodes corresponding to those in panel A are labeled by their ASTRAL node IDs. **(C)** Gene and site concordance at three conflict-prone nodes (N47, N52, N61). Gene concordance factors (gCF) and discordance factors (gDF1, gDF2, and gDFP) are shown on the left. The main topology is shown in dark blue; alternatives and paraphyly are shown in lighter blue shades. Site concordance (sCF) and discordance (sDF1, sDF2) are shown on the right. The red dashed line at 33.3% represents the expected support under a hard polytomy. **(D)** ASTRAL quartet support for the corresponding nodes (N28, N23, N16). Bar plots show the normalized frequency of each of the three possible quartet topologies, with the main topology shown in dark green and the two alternatives in lighter shades. Local posterior probabilities (LPP) are indicated above each bar. In all cases, ASTRAL strongly supports the main topology (LPP = 1.0), despite the presence of gene tree discordance in the ML-based analyses (panel C). This highlights how coalescent-based methods can recover consistent species relationships even in the presence of substantial incomplete lineage sorting, which likely underlies the low gCF and high gDFP values observed at these nodes.

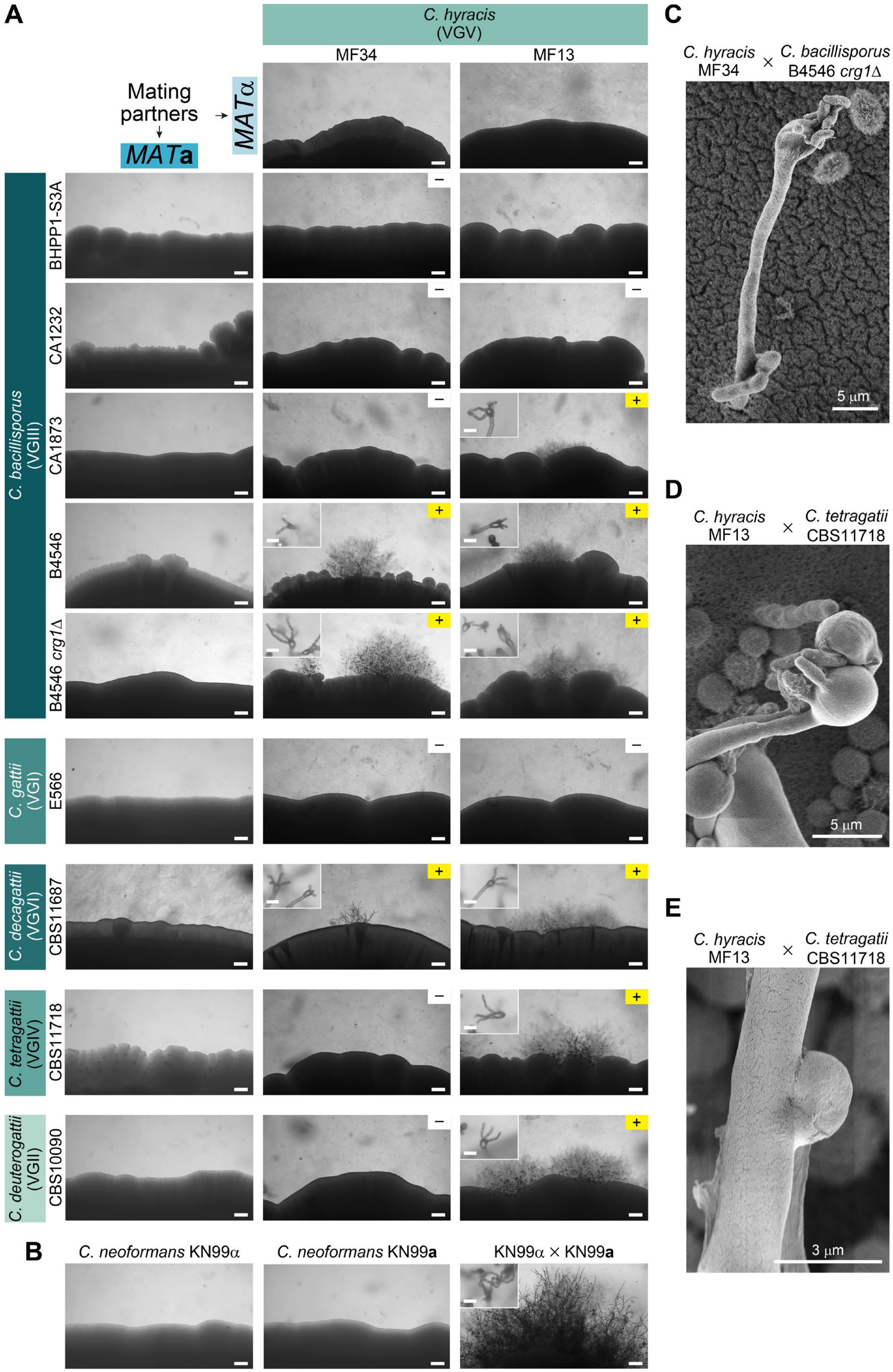
A
C. hyracis
(VGV)
MF34
MF13
Mating partners
MATα
MATa
C. bacillisporus (VGIII)
BHPP1-S3A
CA1232
CA1873
B4546
B4546 crg1Δ
C. gattii (VGI)
E566
C. decagattii (VGVI)
CBS11687
C. tetragattii (VGIV)
CBS11718
C. deuterogattii (VGII)
CBS10090
B
C. neoformans KN99α
C. neoformans KN99a
KN99α × KN99a
C
C. hyracis MF34
×
C. bacillisporus B4546 crg1Δ
5 μm
D
C. hyracis MF13
×
C. tetragatii CBS11718
5 μm
E
C. hyracis MF13
×
C. tetragatii CBS11718
3 μm

47

S2 Fig. Mating assays demonstrate the ability of *Cryptococcus hyracis* to undergo sexual reproduction. **(A)** Pairwise mating assays using two *C. hyracis* *MAT*α strains (MF34 and MF13) crossed with *MAT***a** strains from other species in the *C. gattii* complex. Crosses were performed on V8 agar and examined by light microscopy after 2–3 weeks. Hyphal growth with basidia and spores (inset) indicated successful mating; positive and negative interactions are marked with "+" and "–", respectively. Strain MF13 generally showed stronger mating responses than MF34. For *C. bacillisporus*, we tested both the wild-type strain B4546 and a derived *crg1*Δ mutant. *CRG1* encodes a negative regulator of pheromone signaling that acts via G-protein signaling by stimulating GTP hydrolysis to GDP, thereby extinguishing Gα-GTP signaling. Thus, deletion mutants are hypersensitive to mating signals and serve as sensitive indicators of mating potential. Scale bars = 200 μm (10 μm in insets). **(B)** Positive control cross between *C. neoformans* *MAT*α (KN99α) and *MAT***a** (KN99**a**) showing robust filamentation and basidia formation. **(C–E)** Scanning electron microscopy (SEM) of interspecies mating interactions involving *C. hyracis*. Panels show examples of basidia and basidiospore formation, as well as fused clamp connections, observed in crosses with *C. bacillisporus* (C) and *C. tetragattii* (D–E). These structures confirm the ability of *C. hyracis* to engage in sexual development.

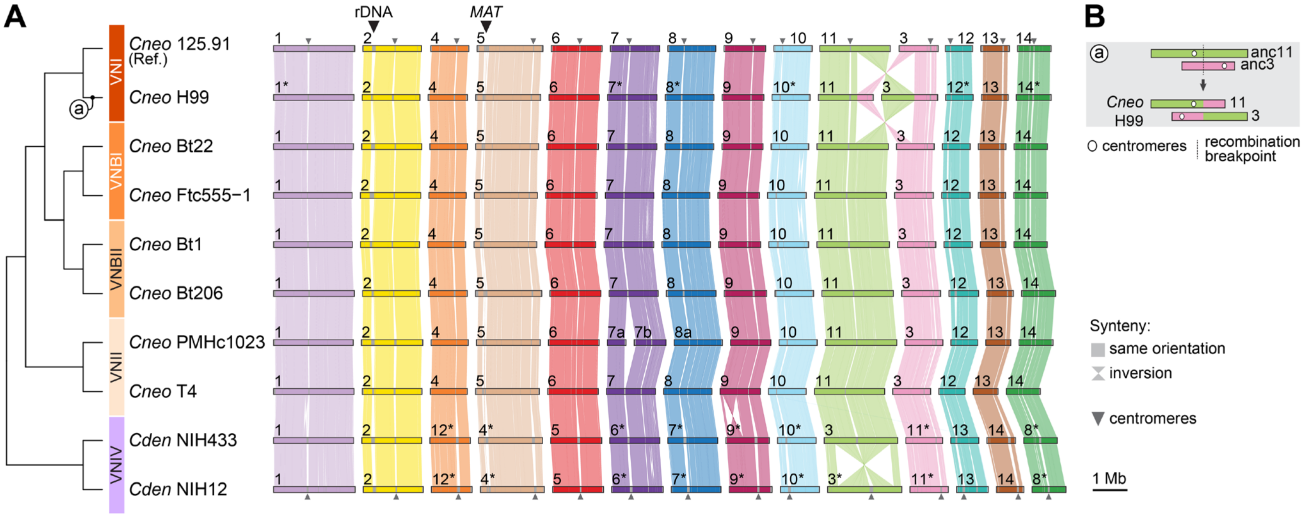

S3 Fig. Karyotype comparisons among *C. neoformans* and *C. deneoformans* strains. (A) Synteny analysis of 14 chromosomes across multiple *C. neoformans* strains representing the four major lineages (VNI, VNII, VNBI, VNBII) and two *C. deneoformans* (VNIV) strains. Chromosomes were ordered to maximize collinearity with the *C. neoformans* strain 125.91 reference genome, and those inverted relative to their original orientations are indicated with asterisks. Overall synteny is well conserved across *C. neoformans* strains, except for strain H99 (VNI), which harbors a known translocation between chrs. 3 and 11. *C. deneoformans* strains are largely syntenic to *C. neoformans*, except for some large inversions. The locations of the rDNA cluster and MAT locus are indicated. Centromere positions are marked by grey triangles. Rearrangements, including large inversions and translocations, were identified using the ntSynt toolkit and visualized with ntSynt-viz. **(B)** Schematic of the previously characterized reciprocal translocation in *C. neoformans* H99 between chrs. 3 and 11. Dashed lines indicate the breakpoint positions, and grey triangles mark centromere locations. This rearrangement distinguishes H99 from other VNI strains.

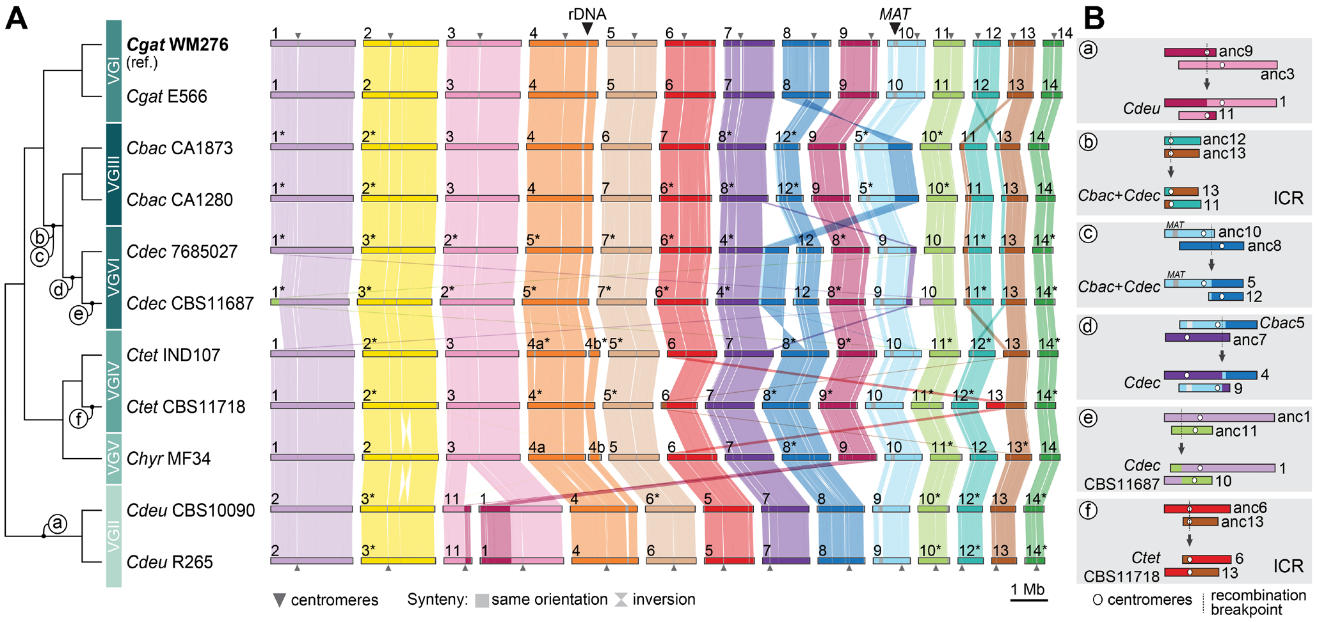

S4 Fig. Karyotype comparisons among *C. gattii* (sensu lato) species. (A) Synteny comparisons between *C. gattii* strain WM276 (VGI, reference) and representative strains from each of the species within the *C. gattii* complex. Chromosomes were reordered to maximize collinearity with the reference, and those inverted relative to their original assembly orientations are marked with asterisks. Synteny blocks were identified using the ntSynt toolkit and visualized with ntSynt-viz. Despite broad conservation of synteny, numerous rearrangements are observed across species, including translocations and inversions. The chromosomal locations of the rDNA cluster and the *MAT* locus are also indicated, as are predicted centromeres (triangles). **(B)** Schematic representations of selected interspecific chromosomal rearrangements. Colored bars represent putative ancestral chromosomes and their rearranged derivatives in extant species, with breakpoints indicated by dashed lines. Centromeres are shown as circles. Panels highlight key rearrangements across different nodes of the phylogeny, including several likely intercentromeric recombination (ICR) events, such as that shared between *C. bacillisporus* and *C. decagattii* (event b) and the derived translocation found in *C. tetragattii* CBS11718 (event f).

A V8 pH 5.0, room temperature, dark, 1 month

	a2 b2 NCYC1536	a2 b4 DSM117830
a1 b1 DSM108351	–	–
a1 b3 DSM108352	+?	–
a1 b3 DSM111204	–	–
a1 b3 DSM111205	–	–

C MS, room temperature, dark, 1 month

	a2 b2 NCYC1536	a2 b4 DSM117830
a1 b1 DSM108351	–	–
a1 b3 DSM108352	–*	–
a1 b3 DSM111204	–	–
a1 b3 DSM111205	–	–

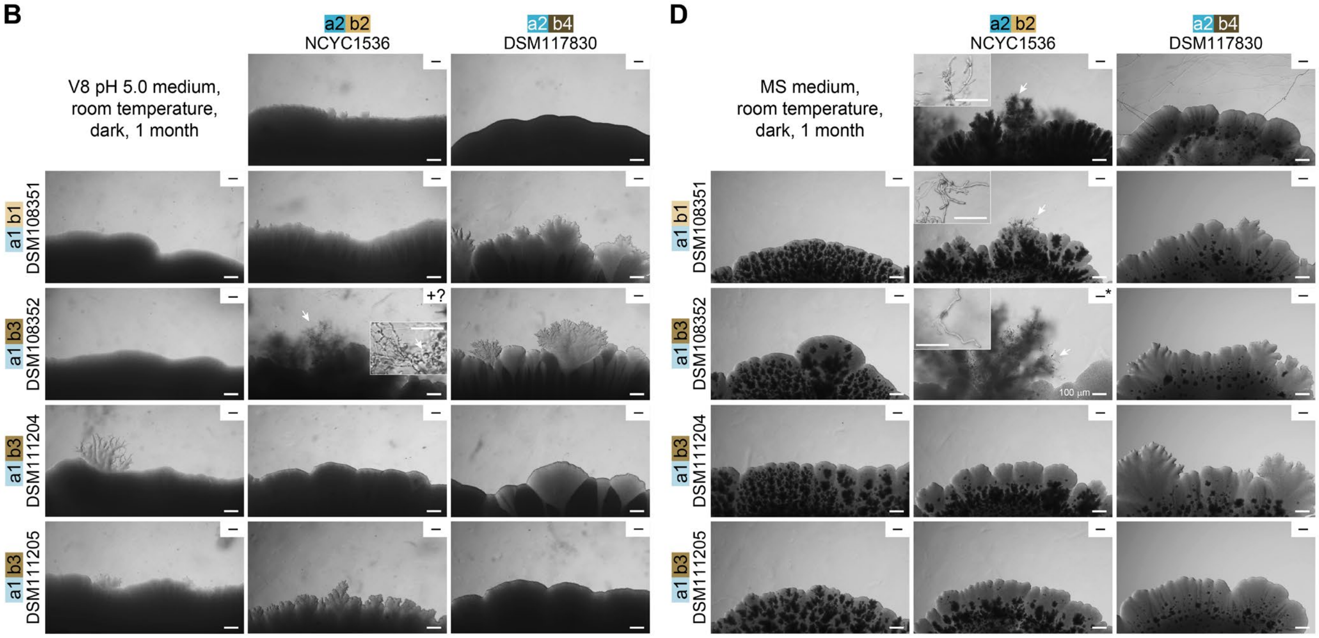

S5 Fig. Mating assays between *C. porticicola* strains under two different media conditions. (A) Summary table indicating presence (+?) and absence (–) of hyphal and basidial-like structures. "+?" denotes putative structures consistent with sexual development, though the identity of basidia-like structures could not be definitively confirmed. Mating-type alleles (*STE3* and *HD*) are shown for each strain. **(B)** Representative light microscopy images from V8 pH 5.0 medium assays. Only the DSM108352 (a1b3) × NCYC1536 (a2b2) cross showed hyphal growth and potential sexual structures embedded in the agar (arrow); the inset highlights a structure resembling a basidium. Scale bars: 200 µm (50 µm for inset). **(C)** Summary table of mating outcomes on MS medium. **(D)** Representative light microscopy images from MS medium assays. Pseudohyphal growth is observed in the solo culture of NCYC1536 and in the DSM108351 × NCYC1536 cross (arrows). The DSM108352 × NCYC1536 cross showed hyphal growth (arrow) but no apparent basidial-like structures throughout the course of the experiment, and is therefore marked as "–*". Scale bars: 200 µm unless otherwise noted (50 µm for insets). Scale bars: 200 µm unless otherwise noted (50 µm for insets).

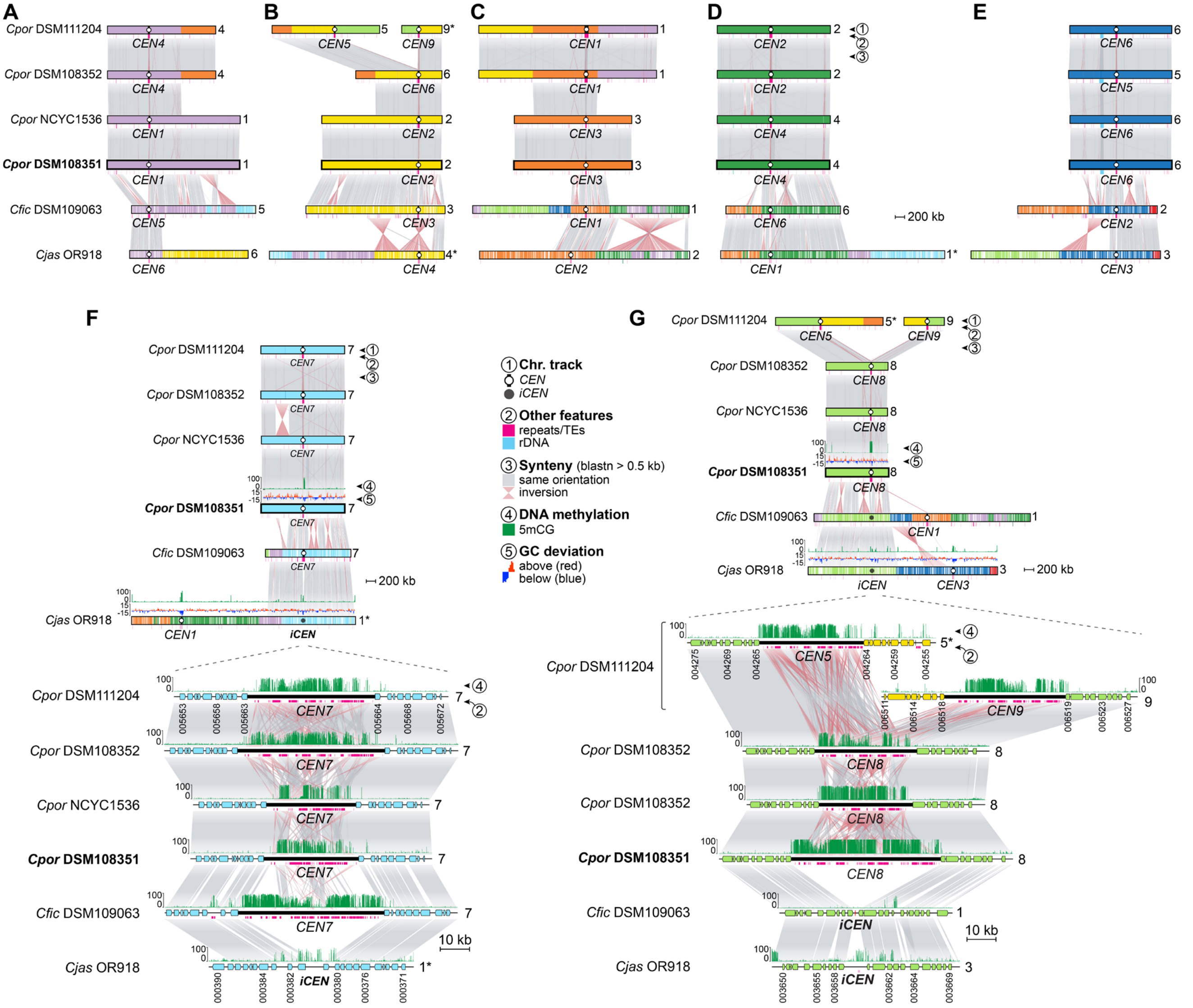

S6 Fig. Synteny and structural context of conserved and lost centromeres across clade D *Cryptococcus* species. (A–E) Synteny comparisons across conserved centromeric regions in *C. porticicola* (strains DSM111204, DSM108352, NCYC1536, and DSM108351), *C. ficicola* (DSM109063), and *C. jaspeensis* (OR918). Tracks highlight conserved centromere positioning and surrounding synteny for *CEN1*, *CEN2*, *CEN3*, *CEN4*, and *CEN6* of *C. porticicola* DSM108351 and their corresponding orthologous regions in the other strains and species. **(F)** Loss of *CEN7* in *C. jaspeensis* OR918. The top panel shows chromosome-scale synteny across strains, highlighting the conserved position of *CEN7* in all species except *C. jaspeensis*, where the orthologous region lacks canonical centromeric structure and is marked as inactivated (*iCEN*). The bottom panel provides a zoomed-in view of this region with tracks showing synteny, 5mCG DNA methylation, and TE content. Although the centromeric repeat landscape is disrupted in OR918, residual DNA methylation and partial synteny may indicate a recently inactivated centromere. **(G)** Example of centromere loss in both *C. ficicola* and *C. jaspeensis*. The top panel shows chromosome-scale synteny, highlighting the position of *CEN8* from *C. porticicola* DSM108351. In strain DSM111204, this region underwent intercentromeric recombination event, while in *C. ficicola* and *C. jaspeensis* the corresponding centromeric region was lost. The bottom panel shows a zoomed-in view of this region, indicating that the orthologous loci in DSM109063 and OR918 lack the repeat-rich structure characteristic of active or ancestral centromeres, and only residual 5mCG DNA methylation, consistent with inactivated centromeres (*iCEN*).

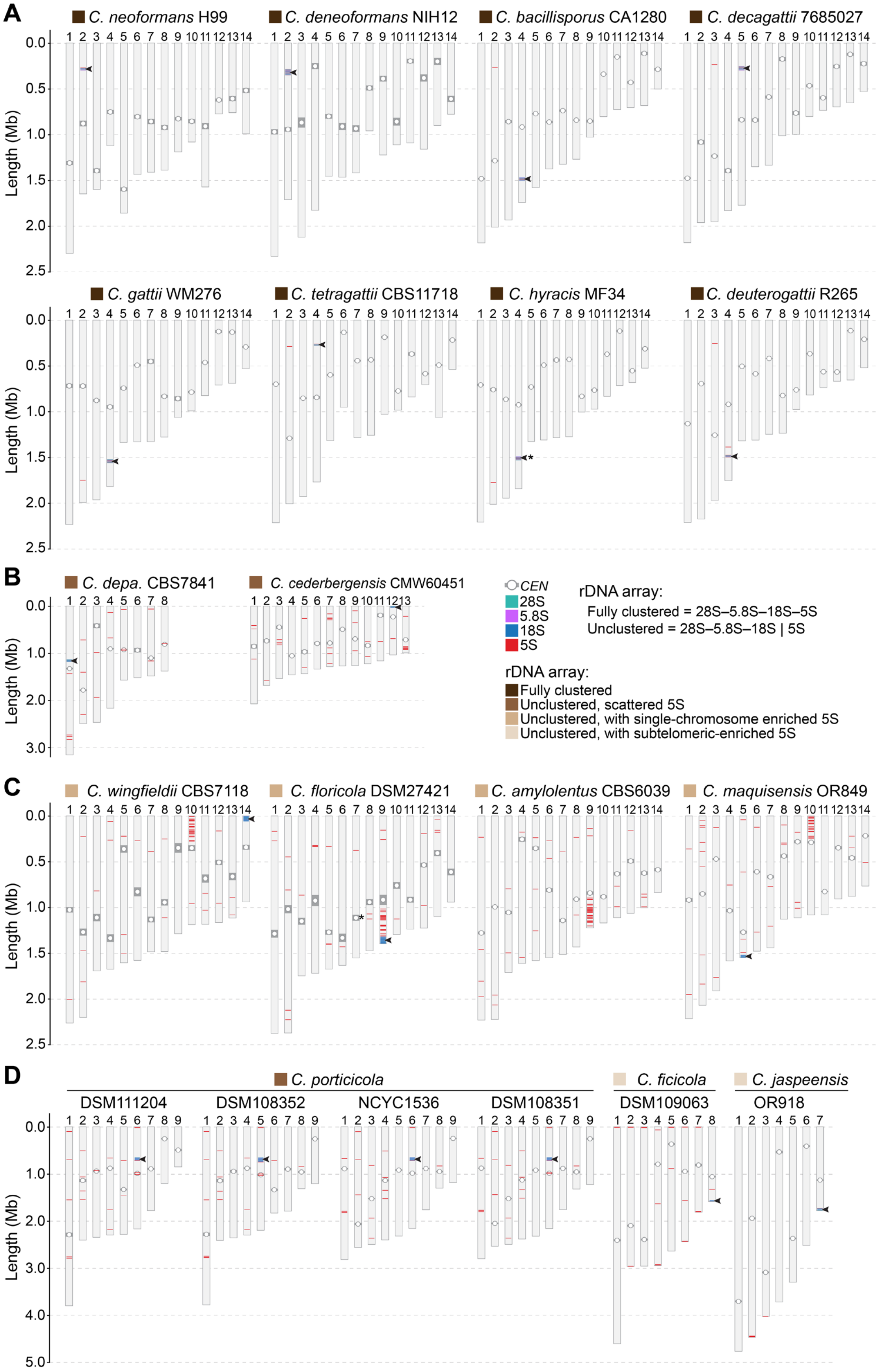

A
C. neoformans H99
C. deneoformans NIH12
C. bacillisporus CA1280
C. decagattii 7685027
C. gattii WM276
C. tetragattii CBS11718
C. hyracis MF34
C. deuterogattii R265
Length (Mb)
B
C. depa. CBS7841
C. cederbergensis CMW60451
CEN
28S
5.8S
18S
5S
rDNA array:
Fully clustered = 28S–5.8S–18S–5S
Unclustered = 28S–5.8S–18S | 5S
rDNA array:
Fully clustered
Unclustered, scattered 5S
Unclustered, with single-chromosome enriched 5S
Unclustered, with subtelomeric-enriched 5S
C
C. wingfieldii CBS7118
C. floricola DSM27421
C. amylolentus CBS6039
C. maquisensis OR849
D
C. porticicola
DSM111204
DSM108352
NCYC1536
DSM108351
C. ficicola
DSM109063
C. jaspeensis
OR918

S7 Fig. Genome-wide organization of ribosomal RNA (rRNA) genes across *Cryptococcus* species. Chromosomal distribution of 28S, 5.8S, 18S, and 5S rRNA genes across all analyzed *Cryptococcus* strains with telomere-to-telomere assemblies. Arrowheads indicate the position of the core rDNA array, and predicted centromeres are marked with open circles. The rDNA array (28S–5.8S–18S) is typically clustered on a single chromosome, whereas 5S genes show variable distribution patterns across lineages. **(A)** Clade A (pathogenic) species exhibit fully clustered rRNA arrays, while saprobic species from other clades **(B-D)** display unclustered configurations with lineage-specific features: scattered 5S genes (e.g., *C. depauperatus* and *C. porticicola*), chromosome-specific enrichment (e.g., *C. floricola*), or subtelomeric localization (e.g., *C. jaspeensis*).

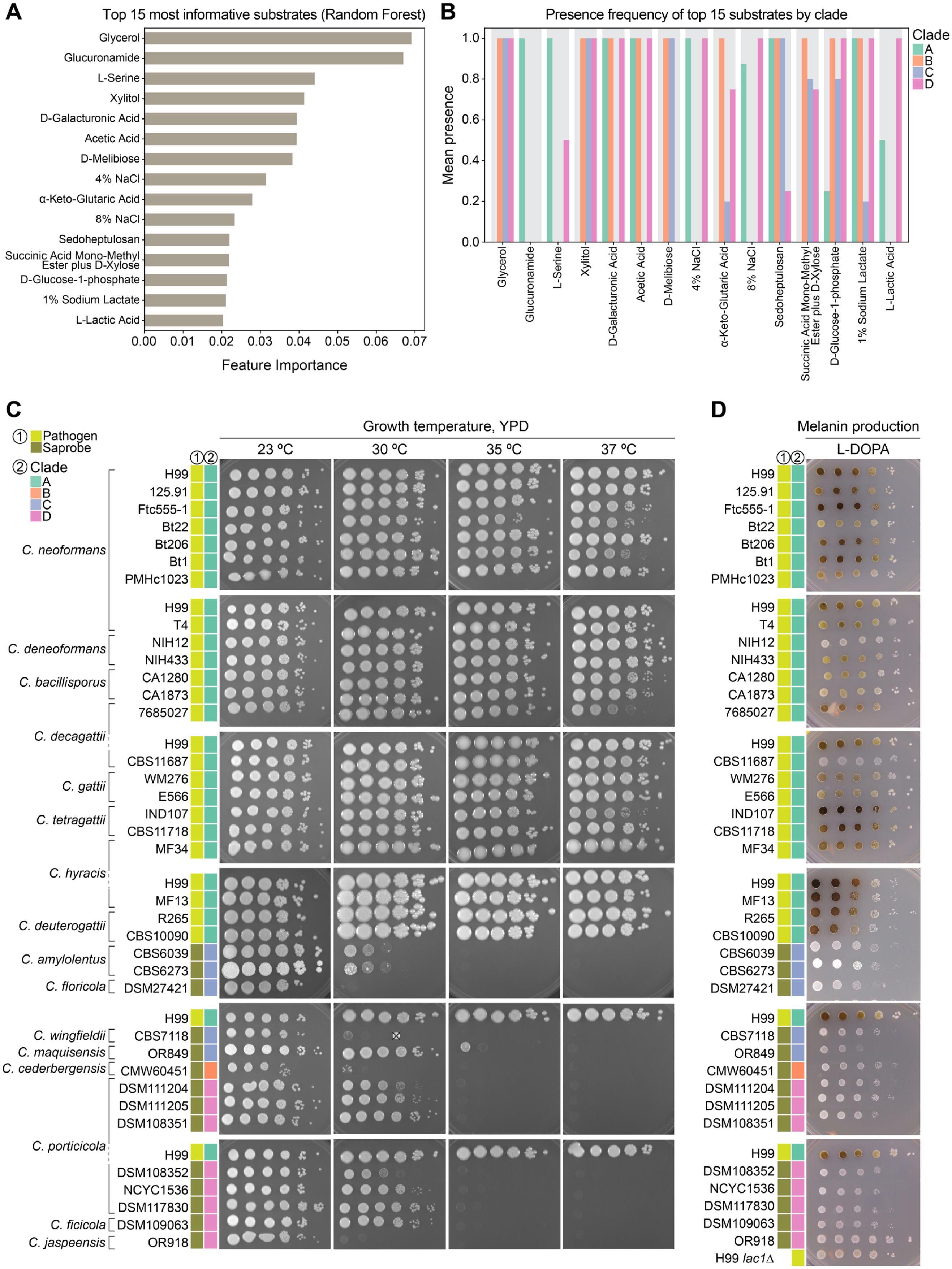
A
Top 15 most informative substrates (Random Forest)
Glycerol
Glucuronamide
L-Serine
Xylitol
D-Galacturonic Acid
Acetic Acid
D-Melibiose
4% NaCl
α-Keto-Glutaric Acid
8% NaCl
Sedoheptulosan
Succinic Acid Mono-Methyl Ester plus D-Xylose
D-Glucose-1-phosphate
1% Sodium Lactate
L-Lactic Acid
0.00 0.01 0.02 0.03 0.04 0.05 0.06 0.07
Feature Importance
B
Presence frequency of top 15 substrates by clade
Mean presence
0.0 0.2 0.4 0.6 0.8 1.0
Clade
A
B
C
D
C
1 Pathogen
Saprobe
2 Clade
A
B
C
D
Growth temperature, YPD
23 ºC
30 ºC
35 ºC
37 ºC
C. neoformans
H99
125.91
Ftc555-1
Bt22
Bt206
Bt1
PMHc1023
T4
C. deneoformans
NIH12
NIH433
C. bacillisporus
CA1280
CA1873
7685027
C. decagattii
CBS11687
C. gattii
WM276
E566
C. tetragattii
IND107
CBS11718
MF34
C. hyracis
MF13
C. deuterogattii
R265
CBS10090
C. amylolentus
CBS6039
CBS6273
C. floricola
DSM27421
C. wingfieldii
CBS7118
C. maquisensis
OR849
C. cederbergensis
CMW60451
DSM111204
DSM111205
DSM108351
C. porticicola
DSM108352
NCYC1536
DSM117830
C. ficicola
DSM109063
C. jaspeensis
OR918
D
Melanin production
L-DOPA
H99 lac1Δ

S8 Fig. Additional phenotypic traits distinguishing *Cryptococcus* species and clades. **(A)** Top 15 phenotypic tests contributing most to clade-level classification based on Random Forest analysis of binarized Biolog data. Substrates are ranked by feature importance in predicting clade membership. **(B)** Mean presence frequency of the top 15 substrates from panel A across *Cryptococcus* clades. Some substrates (e.g., glycerol, xylitol, glucuronamide) display clade-specific patterns, with utilization largely restricted to or absent from particular clades, highlighting their potential utility for phenotypic discrimination. **(C)** Growth of all tested strains at 23 °C, 30 °C, 35 °C, and 37 °C on YPD medium for 4 days. Robust growth at 37 °C was observed exclusively in clade A strains, consistent with thermotolerance as a pathogenicity-associated trait. No strains from saprobic species in clades B–D showed appreciable growth at this temperature. Among saprobes, *C. cederbergensis* displayed limited growth above 30 °C, whereas *C. maquisensis* OR849 exhibited weak growth at 35 °C (visible in the highest inoculum spot). A colony marked with an "X" represents contamination and should be disregarded. **(D)** Melanin production on L-DOPA agar plates incubated at room temperature for 5 days. All clade A strains produced visible melanin, while all nonpathogenic strains from clades B–D lacked pigmentation under these conditions. Among pathogens, melanin production was somewhat reduced in *C. decagattii* CBS11687 and *C. deneoformans* NIH12 under this growth condition.

Supplementary Tables legends

S1 Table. List of strains used in this study, and summary of genome assembly statistics and other genomic features.

S2 Table. ANI, AAI, and dDDH metrics across *Cryptococcus* genomes. **(A)** Full matrix of ANI values computed for all pairwise genome comparisons. **(B)** Summary of pairwise ANI values between species. **(C)** Min., max., and mean ANI values calculated between and within clades, based on grouped pairwise comparisons. **(D)** Full AAI values computed for all pairwise genome comparisons. **(E)** Summary of pairwise AAI values between species. **(F)** Min., max., and Mean AAI values calculated between and within clades, based on grouped pairwise comparisons. **(G)** Closest interspecies genome comparisons based on identity values. **(H)** Digital DNA-DNA hybridization (dDDH) values from Genome-to-Genome Distance Calculator (GGDC). **(I)** dDDH values with species-level summary and strain annotations.

S3 Table. **rRNA gene analysis and statistical tests**. **(A)** Genome-wide summary of rRNA gene counts. **(B)** Genome-wide summary of 5S rRNA gene counts. **(C)** Chromosomal distribution of 5S rRNA genes, observed vs. expected. **(D)** Chromosomal enrichment of 5S rRNA genes and statistical analyses. **(E)** Summary of 5S gene localization relative to subtelomeric regions.

S4 Table. **Biolog phenotype Microarray results**. **(A)** Full list of tests. **(B)** Informative tests showing variable response across strains.

S5 Table. Summary of GlobalFungi ITS matches to newly described *Cryptococcus* species. Table listing exact ITS1 and ITS2 matches identified in the GlobalFungi database for each *Cryptococcus* species. Includes marker resolution (delimitation power) of ITS1 and ITS2 across species, match type

(ITS1 or ITS2), geographic origin, substrate, and study metadata. Data correspond to matches visualized in S1 File.

Table S6. Genome sequencing and information on raw sequencing data generated in this study. (A) Genome sequencing, assembly, polishing, and gene prediction/annotation approaches. **(B)** NCBI accession numbers of each genome and raw read data generated and used in this study.

S1 File. Interactive map of GlobalFungi sampling sites. Interactive HTML map showing GlobalFungi sampling sites with exact ITS1 or ITS2 matches to newly described *Cryptococcus* species, as summarized in S5 Table.
